# A cornichon protein controls polar localization of the PINA auxin transporter in *Physcomitrium patens*

**DOI:** 10.1242/dev.201635

**Published:** 2023-05-05

**Authors:** Carolina Yáñez-Domínguez, Daniel Lagunas-Gómez, Diana M. Torres-Cifuentes, Magdalena Bezanilla, Omar Pantoja

**Affiliations:** ^1^ Departamento de Biología Molecular de Plantas, Instituto de Biotecnología, Universidad Nacional Autónoma de México, Av. Universidad 2001, Cuernavaca, Morelos 62210, México; ^2^ Department of Biological Sciences, Dartmouth, Hanover, NH 03755, USA

**Keywords:** *Physcomitrium patens*, Cornichon, PIN transporter, Secretory pathway, C terminus

## Abstract

Newly synthesized membrane proteins pass through the secretory pathway, starting at the endoplasmic reticulum and packaged into COPII vesicles, to continue to the Golgi apparatus before reaching their membrane of residence. It is known that cargo receptor proteins form part of the COPII complex and play a role in the recruitment of cargo proteins for their subsequent transport through the secretory pathway. The role of cornichon proteins is conserved from yeast to vertebrates, but it is poorly characterized in plants. Here, we studied the role of the two cornichon homologs in the secretory pathway of the moss *Physcomitrium patens*. Mutant analyses revealed that cornichon genes regulate different growth processes during the moss life cycle by controlling auxin transport, with CNIH2 functioning as a specific cargo receptor for the auxin efflux carrier PINA, with the C terminus of the receptor regulating the interaction, trafficking and membrane localization of PINA.

## Teaser

**Summary:** The C terminus of the cornichon CNIH2 protein is important for the plasma membrane localization of the PIN auxin transporter in the moss *Physcomitrium patens*.

## INTRODUCTION

The endomembrane system of eukaryotic cells is a functionally inter-related membrane system composed of many organelles, each with a unique membrane composition where there is a constant exchange of proteins and lipids through a network of membrane trafficking (Bassham et al., 2008; Jürgens, 2004; Kim and Brandizzi, 2014; Morita and Shimada, 2014). Membrane trafficking comprises two main pathways – the secretory and endocytic pathways; both are essential for maintaining a wide range of fundamental cellular functions such as cell proliferation, differentiation, morphogenesis, intercellular communication and signaling, including responses to environmental stimuli (Bassham et al., 2008; Morita and Shimada, 2014). The secretory pathway involves the transport of biosynthetic materials that have been targeted to the endoplasmic reticulum (ER), then flow to the Golgi apparatus (GA) and subsequently to the plasma membrane (PM) or other organelles (Bassham et al., 2008). Newly synthesized membrane proteins in the ER are translocated to the GA by COPII vesicles (Brandizzi and Barlowe, 2013; Kim and Brandizzi, 2014). It is proposed that additional ER membrane proteins, known as cargo receptors, are required for the correct recruitment of membrane proteins to COPII vesicles, as an initial step for their transport to their target membrane. One such family of cargo receptors is the Erv14/Cornichon family protein (Bökel et al., 2006; Castro et al., 2007; Dancourt and Barlowe, 2010; Herzig et al., 2012; Powers and Barlowe, 1998, 2002). Cornichon (Cni) was initially identified in *Drosophila melanogaster*; during oogenesis, Cni is required for the transport of the growth factor α (TGF) Gurken (Grk) to the oocyte membrane. In the absence of *Dm*Cni, oocytes fail to establish adequate dorsoventral symmetry during oogenesis. The loss of *ERV14*, the homolog of cornichon in yeast, causes the formation of a defective budding site due to the inefficient transport of the Axl2p protein, necessary for the establishment of axial polarity, in which ERV14 acts as a cargo receptor, recruiting Axl2p into COPII vesicles (Powers and Barlowe, 1998, 2002).

CNI homolog (CNIH) proteins are also present in plants, but their role in these organisms has been scarcely studied. In rice (*Oryza sativa*) two homologous proteins, *Os*CNIH1 and *Os*CNIH2, have been identified. Using heterologous expression systems in yeast and the epidermis of tobacco leaves (*Nicotiana benthamiana*), it was observed that *Os*CNIH1 localized to the ER and GA, similar to Erv14 in yeast, and that it interacts with the sodium transporter *Os*HKT1;3, suggesting that *Os*CNIH1 functions as a cargo receptor for *Os*HKT1;3 (Rosas-Santiago et al., 2015). In *Arabidopsis thaliana*, five CNIH proteins have been identified, denominated as *At*CNIH1-*At*CNIH5, which are localized to the early secretory pathway; of the five CNIHs, only *cnih1*, *cnih4* and the *cnih1/cnih4* double mutant showed reduced pollen tube tip Ca^+2^ fluxes with a wild-type-like growth rate (Wudick et al., 2018). The *cnih1/cnih4* double mutant affected the correct targeting of the glutamate-like receptors *At*GLR2.2 and *At*GLR3.3, but not other soluble or membrane-attached proteins, suggesting cargo specificity for these two *At*CNIHs. Moreover, *At*CNIH1 and *At*CNIH4 are capable of forming homo- and heteromers, which suggests that the trafficking of *At*GLR depends on the formation of *At*CNIH oligomers (Wudick et al., 2018). From this evidence, it is proposed that CNIHs function as cargo receptors for a variety of PM proteins; and in plants, they appear to play a similar role as their animal and yeast counterparts (Rosas-Santiago et al., 2015; Wudick et al., 2018).

The plant-specific family of PIN-FORMED (PIN) auxin efflux transporters are integral membrane proteins, and some of them are polarly localized to the PM (Zazímalová et al., 2010), having an important role in regulating cell polarity processes by creating an asymmetric distribution of auxin between cells and throughout the plant (Berleth and Sachs, 2001; Friml et al., 2004; Leyser, 2011; Sauer et al., 2006; Wisniewska et al., 2006). In *A. thaliana*, it is established that PIN polar localization and maintenance at the PM is under the control of endocytosis, polar recycling and restriction of lateral diffusion (Dhonukshe et al., 2007; Kleine-Vehn et al., 2008). PIN proteins are internalized via clathrin-mediated endocytosis and cycled back to the PM via distinct trafficking routes which involve the trans Golgi network (TGN) and early endosomes (EE) (Dhonukshe et al., 2007; Kitakura et al., 2011), mediated mainly by the Brefeldin A (BFA) sensitive-ADP Ribosylation Factor Guanine Nucleotide Exchange Factor (ARF-GEF) GNOM (Geldner et al., 2003; Naramoto et al., 2014; Steinmann et al., 1999). Alternative and independent GNOM via, include GNOM-LIKE1 (GNL1) (Teh and Moore, 2007), the BFA-Visualized Endocytic Trafficking Defective 1 (BEN1) (Tanaka et al., 2009), the Rab-type GTPase BEX5 (also known as RABA1B) and the GEF of Rab GTPase VAN4 (Feraru et al., 2012; Naramoto et al., 2014). Intracellular trafficking of PINs is also achieved by the SORTING NEXIN (SNX) 1 and VACUOLAR PROTEIN SORTING (VPS) 29 subunits of the retromer (Jaillais et al., 2007), which is a multimer composed by SNX1/2, VPS35, VPS29 and VPS26, forming a coat on the cytosolic face of endosomes which mediates the recycling and retrograde transport between endosomes and TGN (Bonifacino and Rojas, 2006). The exocyst complex (SEC3, SEC5, SEC6, SEC8, SEC10, SEC15, EXO70 and EXO84 subunits) is an evolutionary conserved component of the eukaryotic sorting machinery that functions as a tethering for exocytic vesicles upon fusion with the plasma membrane (Fendrych et al., 2013). In *Arabidopsis* root cells, loss of the exocyst subunits EXO70A1 or SEC8 causes defects in recycling PIN1 and PIN2 to the PM, adding another mechanism in the control of PIN targeting to the PM (Drdová et al., 2013). Besides these trafficking mechanisms, little is known about the contribution of the early steps of the secretory pathway in the processing and sorting of *de novo* synthesized PIN proteins.

As CNIH proteins participate in the establishment of cell polarity through the regulation of the traffic of cargo proteins to the PM, it was of particular interest to study whether trafficking of PIN proteins occurred through their interaction with the cargo receptor CNIH. Two factors have complicated functional studies of CNIH and PIN proteins in plants. First, plants have expanded CNIH and PIN gene families (Viaene et al., 2014). Second, CNIH proteins in plants have been studied using primarily heterologous expression systems. We used the moss *Physcomitrium patens* to study CNIH-dependent cell trafficking mechanisms for several reasons. Most *P. patens* tissues are a single cell layer thick, enabling single-cell analysis within a tissue context (Cove et al., 2006; Naramoto et al., 2022; Reski, 1998). Unlike other model plants, *P. patens* presents a predominant haploid gametophytic phase and its high frequency of homologous recombination easily enables functional analysis of genes of interest (Kamisugi et al., 2006; Rensing et al., 2008). Here, we characterized the two CNIH genes present in *P. patens* by generating single and double mutants. We found that CNIHs have pleiotropic effects at the gametophytic stage. We analyzed the function of moss CNIH in the early secretory pathway using green fluorescent protein-tagged proteins and confocal microscopy and demonstrated, using protein-protein interaction assays, that the auxin efflux transporter homolog PINA is a cargo protein of the receptor CNIH2; in addition, we identified and characterized the role of C terminus domains in CNIH2 as important for the interaction and polar localization of PINA in protonema cells.

## RESULTS

### Moss cornichon proteins are conserved and possess a longer C terminus

**Fig. 1.**
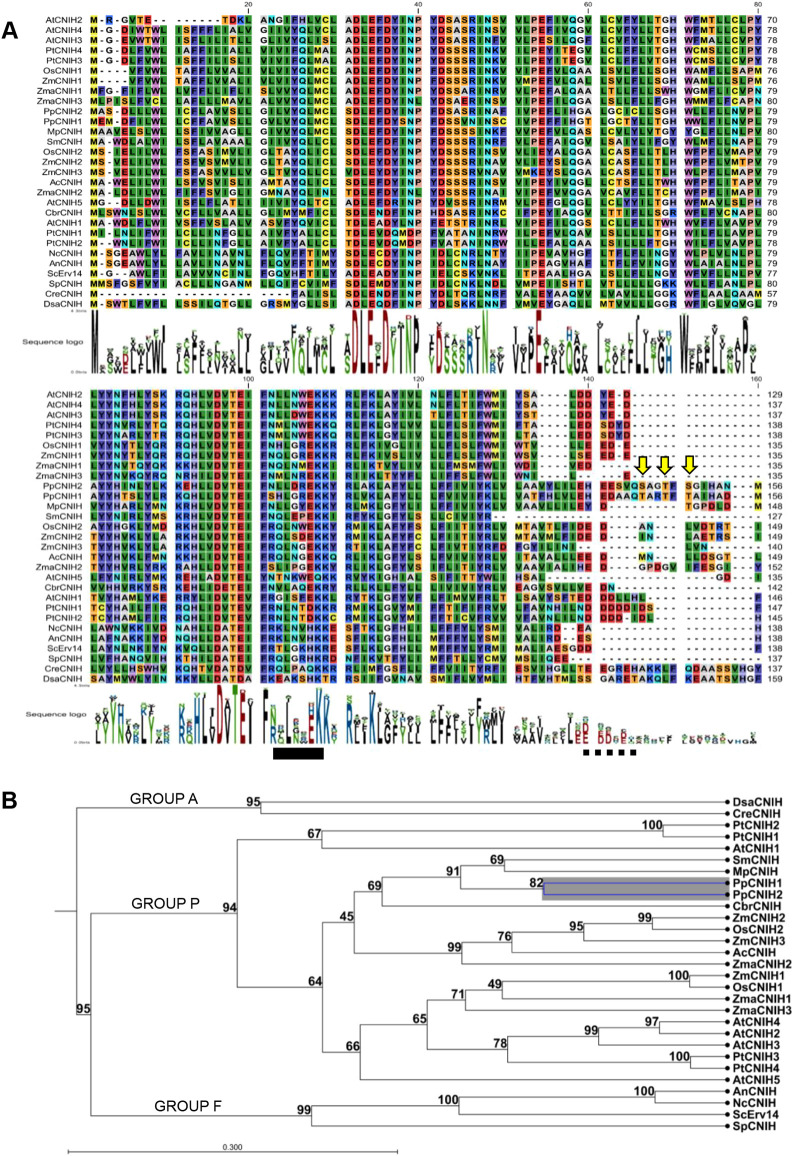
**Multiple amino acid sequence alignment of cornichon homolog proteins and putative phosphorylation sites in moss homologs.** (A) Amino acid sequence alignment of cornichon homologs from algae, plants and fungi; black bar shows the consensus motif IFNXL and dashed black bar the acidic motif (Ac. Dom). Arrows indicate predicted phosphorylation sites on Ser and/or Thr residues identified by the NetPhos3.1 prediction server. (B) Phylogenetic tree of cornichon homologs. Group A is represented by algae; Group P corresponds to angiosperms; and Group F corresponds to fungi. Numbers correspond to bootstrap values (%). The data were analyzed with the software CLC Main Workbench 8.1. For nomenclature details see Materials and Methods. A full-size image of A is shown in Fig. S1.

The cornichon family of proteins is present in all eukaryotes; however, according to previous reports, cornichon proteins from plants and fungi are more similar than homolog proteins from animals (Nakagawa, 2019; Rosas-Santiago et al., 2017). This family of proteins play a role as cargo receptors, including in plants, as previously reported for the angiosperm rice *Os*CNIH1 (Rosas-Santiago et al., 2015) and *Arabidopsis At*CNIH1-4 (Wudick et al., 2018). In view of this evidence, we wanted to know whether cornichon proteins were also present in the bryophyte *P. patens*. Using BLAST analysis we identified two *P. patens* cornichon genes, *CNIH1* (Pp3c11_17020V3.1) and *CNIH2* (Pp3c7_11500V3.1), with homology to algae, plants and fungi proteins (Fig. 1A); each gene encodes a protein that is 156 amino acids in length. We readily identified the IFXXL sequence motif in CNIH1 and CNIH2, which is similar to the IFRTL domain (IFX/NL in plants) (Fig. 1A, solid bar), that serves as an interaction site with the COPII component *Sc*SEC24p in yeast (*Saccharomyces cerevisiae*) (Pagant et al., 2015; Powers and Barlowe, 2002). Both moss proteins also possess the acidic domain that has been reported to be restricted to plant and fungal homologs that participates as a binding site for cargo proteins (Rosas-Santiago et al., 2017) (Fig. 1A, dashed bar). Interestingly, compared with other homologs, *P. patens* CNIH proteins possess an extended C terminus with 15 extra amino acids, characterized by the presence of several putative phosphorylation residues (Fig. 1A, arrows and Fig. S1). According to the phosphorylation prediction server NetPhos3.1 (Blom et al., 1999), in CNIH1 the three threonine residues (T145, T148 and T150) are potential phosphorylation residues (Fig. S2A); however, for CNIH2, only T148 is predicted as a potential phosphorylation site (Fig. S2B). We also analyzed the evolutionary relationship of moss CNIH with algae, plants and fungi homologs using the UPGMA algorithm (Fig. 1B). Cornichon proteins are grouped into three main categories (Fig. 1B); in Group A we exclusively found cornichon homologous from chlorophyte algae; the second group corresponds to higher plants (Group P), and the third group is composed of fungal proteins (Group F).Together, these results indicate that, in general, moss cornichon proteins are as conserved as their homologs in plants, suggesting that they could play a similar function as cargo receptors in *P. patens*.

### Mutations of cornichon homologs cause subtle morphological changes along the life cycle of the moss

**Fig. 2.**
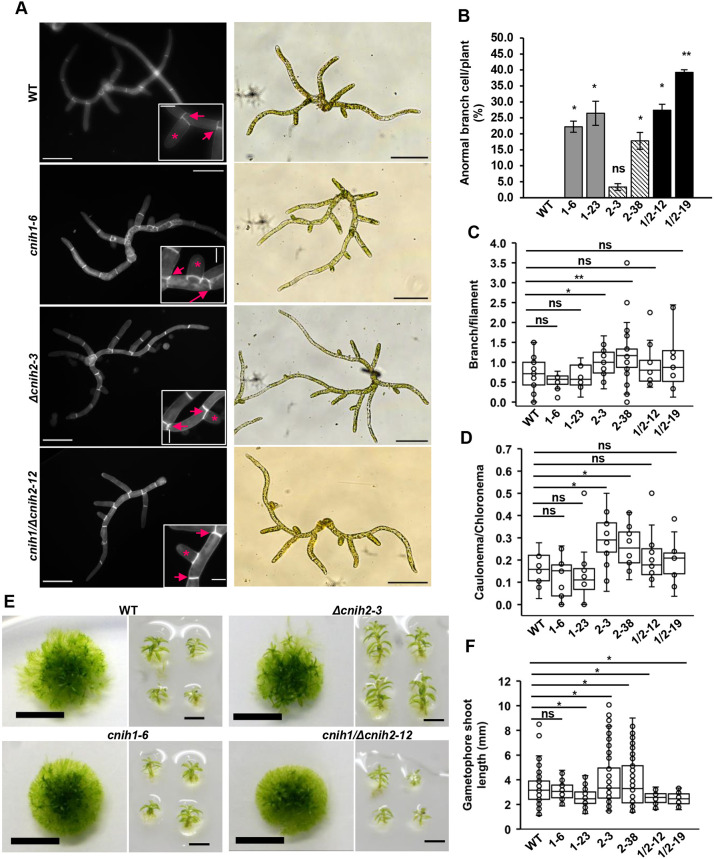
**Cornichon mutants have pleiotropic effects during the gametophyte moss life cycle.** (A) (Left column) Protonema from WT and mutant lines stained with Calcofluor White after 7 days of growth, visualized under epifluorescence microscopy. Insets show apical ends of cells (arrows) and abnormal lateral cell at middle position (asterisk). (Right column) Brightfield images from 7-day-old protonema; auto-contrast was used in all images. Scale bars: 100 µm; insets 20 µm. (B) Percentage of anormal lateral cells emerging in the middle in protonema by plant at 7 days. *n*≥26. Error bars indicate s.d. ns, *P*≥0.05; *P≤0.05; **P≤0.001. (C) Branching ratio of protonema lines measured by quantifying the number of total lateral cells divided by the total cell number per filament at 7 days. *n*≥11. ns, *P*≥0.05; *P≤0.05; **P≤0.001. (D) Calculated caulonema/chloronema ratio. *n*>12. ns, *P*≥0.05; *P<0.05; **P≤0.001 (unpaired two-tailed *t*-test was performed for protonema statistics, data from at least two experimental repeats). (E) Colony (left) and individual gametophores (right) from WT and mutant lines at 4 weeks of growth. Scale bars: 5 mm, left; 2 mm, right. (F) Gametophore shoot length. *n*≥59. ns, *P*≥0.05; *P<0.05 (ANOVA and Tukey-Kramer post hoc test, data from three experimental repeats). Box plots show the first and the third quartile, and the median (horizontal line); whiskers show the minimum and the maximum values. WT, wild type; 1-6, *cnih1-6*; 1-23, *cnih1-23*; 2-3, Δ*cnih2-3*; 2-38, Δ*cnih2-38*; 1/2-12, *cnih1/*Δ*cnih2-12*; 1/2-19, *cnih1/*Δ*cnih2-19.*

To understand the physiological role of the moss cornichon genes, we used the CRISPR-Cas9 system (Mallett et al., 2019) to edit *CNIH1*, resulting in an in-frame premature stop codon at nucleotide position 132, which would encode a 42 amino acid peptide (*cnih1*; Fig. S3). For *CNIH2*, the null mutant (Δ*cnih2*) was generated by homologous recombination, replacing the corresponding locus by a hygromycin resistance cassette (Fig. S4). To obtain the double mutant, we used homologous recombination to replace the *CNIH2* locus with the hygromycin resistance cassette in the *cnih1-23* single mutant (Figs S3 and S4). We analyzed two independent lines of each mutant, from two independent rounds of transformation events. All the mutants were viable and protonemal growth was similar to the wild type (WT) (Fig. 2A; Fig. S5A). However, we found that mutant plants exhibited abnormal branching, with side branch initials forming in the middle of the subapical cell, instead of initiating at the apical end of the subapical cell, as normally observed in WT protonemata (Fig. 2A, asterisks and arrows; Fig. S5A). To corroborate these observations, we quantified the frequency of abnormal branching, observing that ∼22-26% of the branches in the *cnih1* and 3-17% in Δ*cnih2* single mutants showed abnormal branching, whereas no abnormal branching was observed in the WT (Fig. 2B). For the *cnih1/*Δ*cnih2* double mutant, the phenotype was similar to *cnih1*, with 27-39% of the branching cells occurring in the middle of the subapical cell (Fig. 2A,B). Additional alterations in protonemal morphology were observed in the Δ*cnih2* mutant, including an increased number of side branch cells (Fig. 2C) and early development of caulonemal cells, confirmed by the larger calculated caulonemal/chloronemal cell ratio (Fig. 2D). These results suggest that *CNIH1* helps to regulate positioning of the branch initial cells in protonemata; in addition, *CNIH2* seems to play a role in negatively regulating protonemal branching, and in cell differentiation from chloronemata to caulonemata.

Gametophores from the Δ*cnih2* mutant were bigger than those from WT, while those from the *cnih1* and *cnih1/*Δ*cnih2* mutants were smaller than those from WT (Fig. 2E,F; Fig. S5B). Together, these results indicate that CNIH genes are controlling the growth of protonema and gametophores in an opposing way, where *CNIH1* acts as a dominant gene over *CNIH2*, as in the *cnih1/*Δ*cnih2* double mutant the phenotype is more similar to the *cnih1* single mutant (Fig. 2). Among the phenotypes observed from the three cornichon mutants, the early caulonemal development in the Δ*cnih2* mutant was of particular interest because it has been suggested that polar transport of auxins mediated by auxin efflux transporters (PIN) are important for the transition from chloronemata to caulonemata (Bennett et al., 2014; Viaene et al., 2014). Interestingly, deletion of the PINB transporter also generated larger gametophores like in the Δ*cnih2* mutant (Bennett et al., 2014). These similarities led us to explore a possible interplay between moss CNIHs and the PIN transporters.

### CNIH2 is the cargo receptor for the auxin efflux transporter PINA that controls protonemal development

**Fig. 3.**
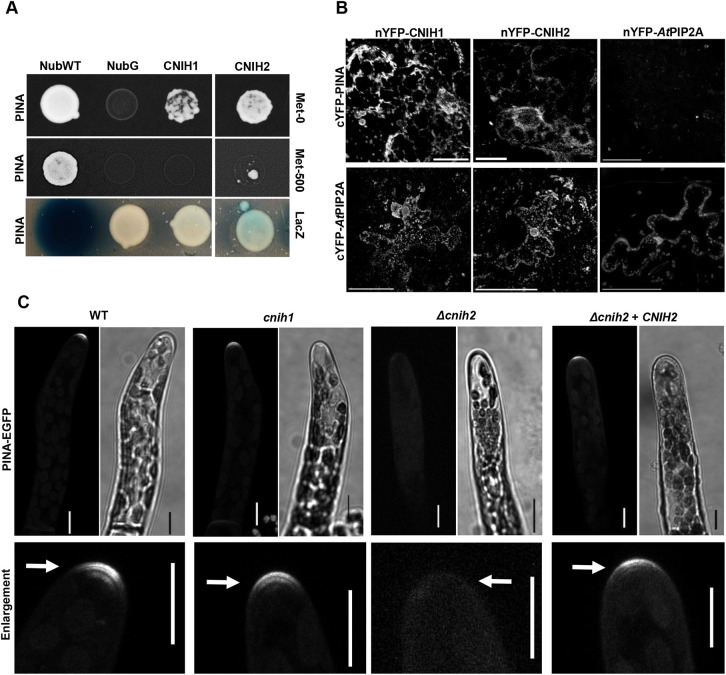
**CNIH2 protein is the cargo receptor for the auxin efflux carrier PINA.** (A) Protein-protein interaction identified by the mbSUS assay with the moss cornichon proteins (Nub fusions) and the auxin transporter PINA (Cub fusion). Yeast cell growth in selection medium (Met-0); the differential interaction was confirmed by cell growth inhibition under repressive selection conditions (Met-500) and by the lower activity of *lacZ* (intensity of the bluish color). NubWT and NubG were used as false negative and false positive controls, respectively. Uncropped image is in Fig. S6A. (B) Interaction between PINA and CNIH1 or CNIH2 was confirmed by reconstitution of split-YFP fluorescence by the co-expression of nYFP-CNIH1/2 with c-YFP-PINA proteins at the ER. Images are ROI from original images in Fig. S6. Scale bars: 10 µm. The lack of interaction between cYFP-PINA and the aquaporin nYFP-*At*PIP2A was indicated by the absence of fluorescence (negative control). Oligomerization of the aquaporin (nYFP-*At*PIP2A and cYFP-*At*PIP2A) was used as positive control. The interaction between the aquaporin cYFP-*At*PIP2A and nYFP-CNIH1/2 was evinced by the reconstitution of YFP fluorescence. Contrast adjustment was used to improve images. Scale bars: 50 µm. (C) Apical localization of PINA-EGFP in WT was unaffected in the *cnih1* mutant but delocalized in the Δ*cnih2* mutant, a phenotype recovered by complementation with *CNIH2*. Bottom panels are ROI enlargements of apical protonema cells from the corresponding EGFP images; arrows indicate PINA-EGFP localization. Images are *z*-projections with the maximal intensity. Scale bars: 10 µm.

To investigate a possible link between cornichon and the PIN transporters, we analyzed protein-protein interactions between the cornichon homologs and one of the auxin transporters, PINA, the main isoform expressed in all moss tissues (Bennett et al., 2014). As PINs and cornichons are integral membrane proteins, we employed the mating-based split ubiquitin system (mbSUS) that is designed to identify interactions between membrane proteins (Lalonde et al., 2010; Obrdlik et al., 2004). We found that CNIH2 interacted more strongly with PINA than CNIH1, as indicated by enhanced growth on selection medium (Fig. 3A, Met-0) and lower inhibition caused by Met (Fig. 3A, Met-500). We also observed greater *lacZ* activity for the interaction between CNIH2 and PINA compared with CNIH1 and PINA (Fig. 3A, *lacZ*). In addition, by employing bimolecular fluorescence complementation (BiFC) in *N. benthamiana* epidermal cells, we confirmed that CNIH1 and CNIH2 interacted with PINA on reticulated structures that resemble the ER, as well as puncta distributed throughout the cytoplasm (Fig. 3B; Fig. S6B). The well-established homo-oligomerization of the aquaporin *At*PIP2 (Maurel et al., 2015) was used as a positive control for the BiFC assay (Fig. 3B). The interaction between cornichon and the aquaporin agreed with similar interactions identified in *Arabidopsis* (Jones et al., 2014). Co-expression of two PM proteins, the auxin transporter and the aquaporin, resulted in no observable fluorescence, indicating that PINA does not interact with the aquaporin and served as a negative control (Fig. 3B). Given the interaction between cornichon and the auxin transporter, we hypothesized that trafficking of the latter might be altered upon cornichon mutation. To test this, we mutated *CNIH1* or *CNIH2* using CRISPR-Cas9 or homologous recombination, respectively, in the moss reporter line PINA-EGFP (Viaene et al., 2014). We obtained two independent lines for each from two different transformations (Figs S3 and S4). Without *CNIH1*, PINA-EGFP localization was not modified (Fig. 3C; Fig. S7A); however, deletion of *CNIH2* resulted in mislocalization of the auxin transporter, as indicated by the absence of the apical fluorescence associated with PINA-EGFP (Fig. 3C; Fig. S7A). To confirm that apical localization of PINA depended on CNIH2, we transiently expressed the full coding sequence of *CNIH2* with the ubiquitin promoter in the Δ*cnih2*/PINA-EGFP mutant line and observed restoration of PINA apical localization (Fig. 3C). These results demonstrate that CNIH2 is required for PINA trafficking to the PM in protonemal cells.

### CNIH2-associated puncta are insensitive to BFA and are associated with a SEC23G subpopulation of ER exit sites

**Fig. 4.**
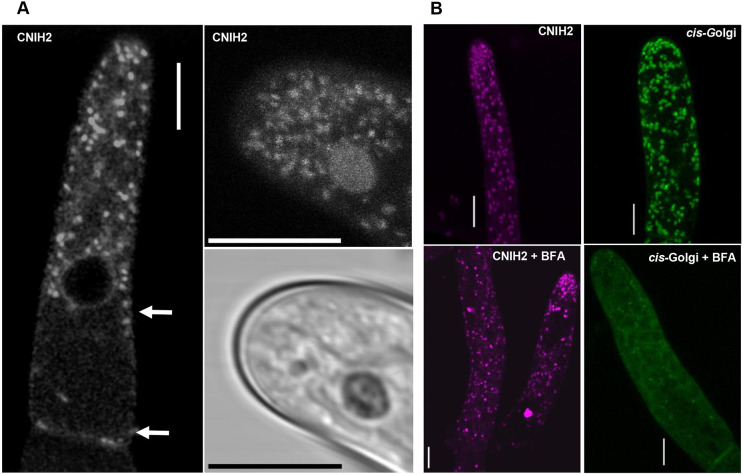
**CNIH2 localizes at the ER and puncta insensitive to BFA.** (A) Subcellular localization of endogenous CNIH2 at the ER and associated puncta in the apical protonemal cell (left) and concentrated at the apex zone (top right); confocal image taken from the *CNIH2-3XmRuby* transgenic moss line; white arrows indicate peripheral localization at ER. Bottom right shows brightfield image of the apex zone. Scale bars: 10 µm. (B) Subcellular localization of CNIH2-3XmRuby (left) and cis-Golgi (YFP-GmMan, right) in an apical protonemal cell before (top) and after (bottom) exposure to 50 µM Brefeldin A for 24 h (+BFA). Scale bars: 10 µm.

**Fig. 5.**
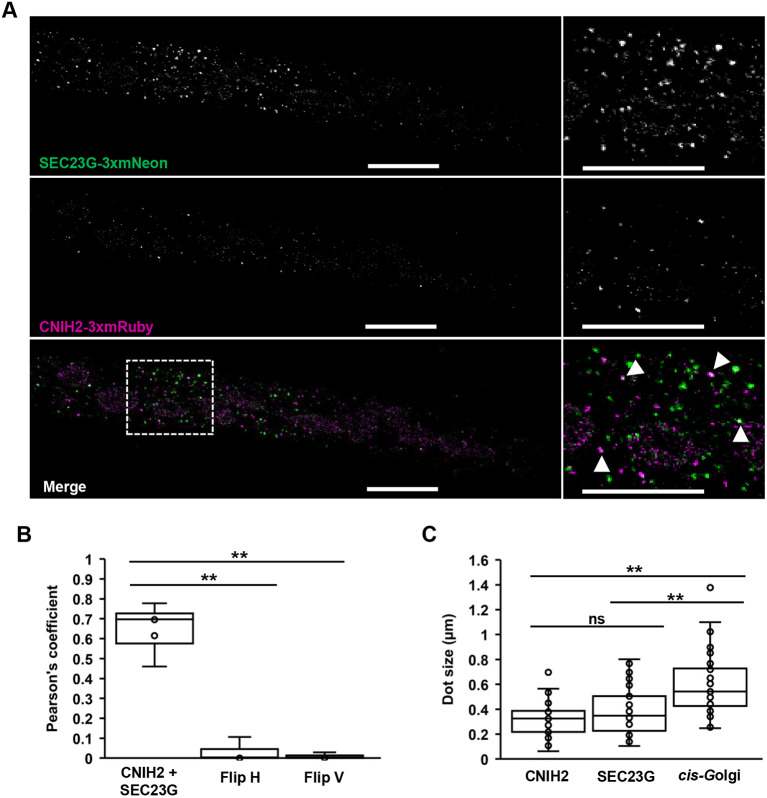
**CNIH2 localizes at SEC23G-confined ERES.** (A) Left panels show localization of endogenous SEC23G (top), CNIH2 (middle) and co-localization of both tagged proteins in a protonemal apical cell (bottom). Right panels show magnification of an ROI (dashed square). Arrowheads show co-localization of both proteins. Representative *z*-projections with maximal intensity confocal image, brightness and contrast were identically enhanced for each image; original images are presented in Fig. S10. Scale bars: 10 µm. (B) Calculated Pearson's correlation coefficient for the co-localization of CNIH2- and SEC23G-tagged proteins in comparison with horizontal (Flip H) and vertical (Flip V) flipped SEC23G images. **P≤0.001 (paired two-tailed *t-*test); *n*=6 cells. (C) Vesicle size for CNIH2 (0.38±0.18 µm), SEC23G (0.32±0.13 µm) and cis-Golgi (0.59±0.24 µm) from protonemal cells; *n*=35. Data are mean±s.d.; ns, *P*≥0.05; **P≤0.001 (paired two-tailed *t*-test). Box plots show the first and the third quartile, and the median (horizontal line); whiskers show the minimum and the maximum values.

The cornichon family of proteins has been characterized as cargo receptors in the ER for the selection of cargo membrane proteins to be transported to the Golgi as part of the early secretory pathway (Castro et al., 2007; Herzig et al., 2012; Rosas-Santiago et al., 2015). To determine the localization of CNIH2 in moss cells, we used CRISPR-Cas9 in combination with homology directed repair (CRISPR-Cas9 & HDR) (Mallett et al., 2019) to insert three tandem sequences encoding for mRuby2 (hereafter, 3XmRuby) in-frame with the coding sequence of *CNIH2* at the endogenous locus (Fig. S8A,C,D). We found that CNIH2-3XmRuby localized to puncta throughout the cell and at the ER. We also observed the puncta localized around the nucleus, which corresponds to perinuclear ER, and a particular accumulation of the puncta near the apex of protonemal cells (Fig. 4A, right). CNIH2-3XmRuby was also localized at the cell periphery in protonemal cells, corresponding to cortical ER (Fig. 4A, left, arrows). This subcellular localization was consistent with what we observed in protonemal cells transiently overexpressing the *ZmUBIpro:*CNIH2-EGFP or *ZmUBIpro:*CNIH1-EGFP fusions (Fig. S7). To identify the organelle associated with the CNIH2-3XmRuby puncta, we treated protonemal cells with BFA, a drug that disassembles the Golgi and causes its redistribution back into the ER (Chardin and McCormick, 1999; Ito et al., 2012; Roberts et al., 2018). Employing the cis-Golgi marker line (YFP-GmMan1), we confirmed that Golgi-associated vesicles were disrupted by BFA (Fig. 4B, right bottom panel). Under this condition, however, CNIH2-3XmRuby puncta were still observed (Fig. 4B, left bottom panel), indicating that they do not correspond/localize to the GA. It is well established that cargo proteins are loaded into ER subdomains known as ER exit sites (ERES), where the COPII subunits Sec23, Sec24, Sec13 and Sec31 are recruited (Brandizzi and Barlowe, 2013). Recently, three protonemal-expressed Sec23 isoforms were shown to associate with the ER, with SEC23G forming larger puncta compared with SEC23B and SEC23D (Chang et al., 2022). The SEC23G puncta were remarkably similar in size to CNIH2-associated puncta. To test for co-localization of CNIH2 and SEC23G in protonemal cells, we employed CRISPR-Cas9 & HDR to insert three tandem sequences encoding for mNeon (hereafter, 3XmNeon) in-frame with the coding sequence of *SEC23G* at the endogenous locus in the *CNIH2-3XmRuby* line (Fig. S8B-D). Using confocal microscopy, we observed partial co-localization of CNIH2-3XmRuby with SEC23G-3XmNeon (Fig. 5A), as indicated by the calculated Pearson's correlation coefficient of 0.66 (Fig. 5B). To corroborate that the co-localization analysis was significant, we flipped horizontally or vertically the Sec23G images and repeated the co-localization analysis and found that the correlation coefficients were significantly lower (Fig. 5B). Coincidentally, the puncta individually highlighted by both proteins shared a similar size, but were smaller than those associated with the Golgi (Fig. 5C). These results suggest that the puncta associated with CNIH2 may correspond to SEC23G-structured ERES and that the polarized localization of moss PINA is mediated by the early secretory pathway through its association with CNIH2.

### The C terminus of CNIH2 regulates its interaction with PINA and trafficking of the transporter

**Fig. 6.**
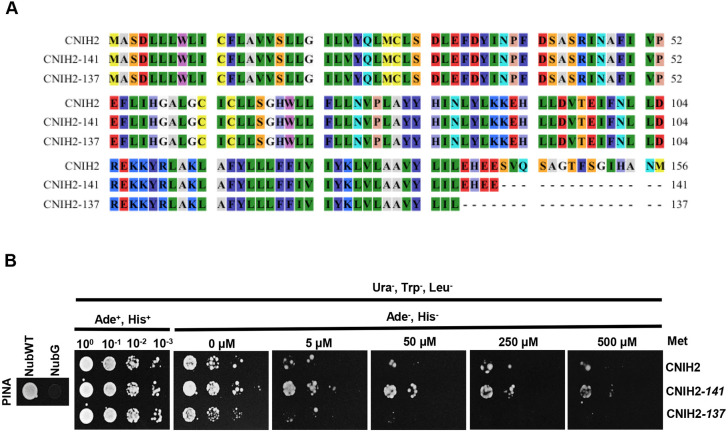
**The C terminus of CNIH2 is important for the protein-protein interaction with PINA.** (A) Amino acid sequence alignment for CNIH2 and truncated C-terminal proteins; CNIH2-141 (T/S domain removed) and CNIH2-137 (T/S and acidic domains removed). Image generated with the software CLC Main Workbench 8.1. (B) mbSUS assay indicated the enhanced interaction of the cargo PINA (Cub fusion) with CNIH2-141 (Nub fusion) and a diminished interaction with CNIH2-137 (Nub fusion) under increasing concentrations of Met, in comparison with CNIH2. NubWT and NubG were used as false negative and false positive controls, respectively. Growth in the presence of Ade and His corresponds to control growth conditions.

**Fig. 7.**
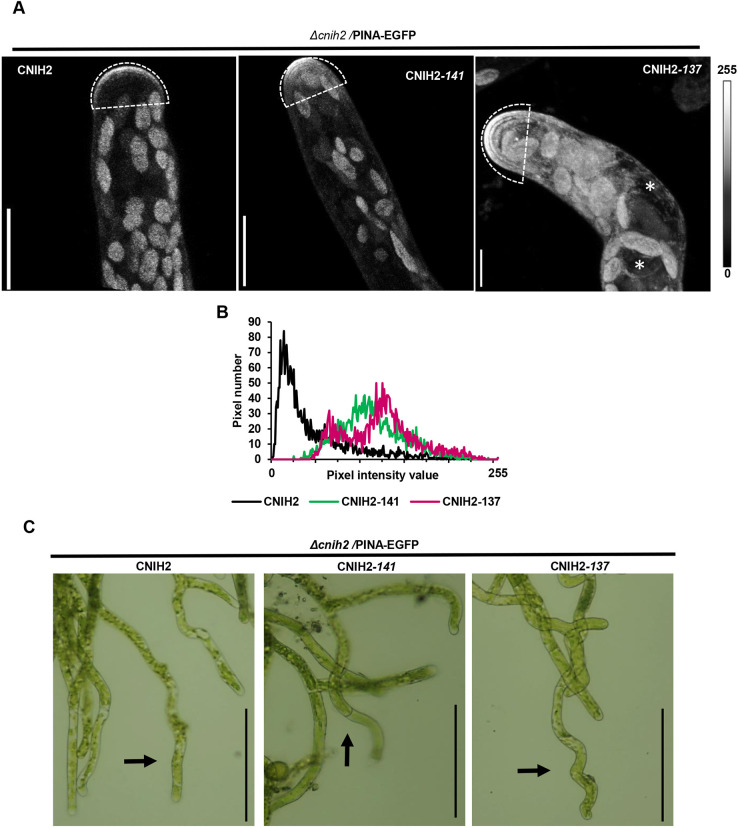
**The C terminus of CNIH2 regulates PINA trafficking in protonema cells.** (A) Correct localization of PINA-EGFP by complementing with the CNIH2 full-length (left) was modified by complementation with the CNIH2-141 (center) and CNIH2-137 (right) truncated proteins, using the mutant Δ*cnih2-3*/PINA-EGFP moss reporter line as genetic background. Confocal images of protonema apical cells show changes in the polar localization of PINA. Images are *z*-projections with maximal intensity. An ROI was delimited (dashed line) at the protonema apex and used for quantification analysis in B. Asterisks shows PINA-EGFP at ER localization. Scale bars: 10 µm. (B) Quantification of fluorescence intensity from the ROIs shown in A. Pixel values 0 to 255 in a gray 8-bit scale. (C) Complementation of mutant Δ*cnih2-3*/PINA-EGFP plants with the full-length CNIH2 showed normal growth; however, when complemented with truncated CNIH2-141 or CNIH2-137 proteins plants generated an undulating protonema (arrows). Scale bars: 100 µm.

Correct trafficking of cargo membrane proteins depends on specific domains present in the Erv14/CNIH protein family (Pagant et al., 2015; Rosas-Santiago et al., 2017), which led us to analyze domains in CNIH2 that might influence PINA trafficking. One of the characteristics of moss CNIH homologs is the presence of a long C terminus with an acidic domain, and a domain containing potential phosphorylation sites (Fig. 1A). To investigate whether these domains regulated CNIH2 function, we generated two truncations, one removing the domain containing the putative phosphorylation site, denoted T/S, at the extreme C terminus (CNIH2-141), and the second removing the T/S and the acidic domain (CNIH2-137) (Fig. 6A). To determine whether the truncations affected CNIH2 interactions with PINA, we employed the mbSUS assay. As shown in Fig. 6B, the strength of the interaction was enhanced when the T/S domain was removed (CNIH2-141), as indicated by cell growth in the presence of 0.5 mM Met but reduced when both domains were removed (CNIH2-137). These results suggest that the T/S domain is involved in regulating the strength or the stability of the interaction with the cargo; however, it remains to be demonstrated whether the phosphorylation state of T148 (Fig. S2B) is indeed involved in this response. These results also demonstrated that the conserved acidic domain at the C terminus is important to maintain the interaction with the cargo, which agrees with previous results from plants and fungi (Rosas-Santiago et al., 2017). Even with a complete C-terminal deletion, the receptor maintained a weak interaction with the cargo (Fig. 6B; 0 µM Met), indicating the possible participation of other interaction sites that have yet to be identified. To corroborate whether the CNIH2 truncations were expressed properly in the moss, we transiently expressed the WT and C-terminal truncations of CNIH2 fused to GFP in moss protoplasts and found that WT CNIH2 and the respective C-terminal truncations were expressed at the ER (Fig. S11). Based on these results and to ascertain the physiological importance of the C terminus of CNIH2, we investigated whether the truncations influenced the localization of PINA by transiently transforming and expressing the coding sequence of WT *CNIH2* or the truncated coding sequences of *CNIH2* driven by the maize ubiquitin promoter in the mutant Δ*cnih2*/PINA-EGFP line. After maintaining plants for 12 days on selection medium, the protonemata from surviving plants were analyzed. The characteristic polarized localization of PINA-EGFP restricted to the tip of the apical cell was reconstituted by transformation with WT *CNIH2* (Fig. 7A, left); however, transformation with *CNIH2-141* resulted in diffuse PINA-EGFP fluorescence that covered a larger area of the tip, and was less restricted to the apex of the apical cell (Fig. 7A, middle). Transformation with *CNIH2-137* resulted in even more diffuse PINA-EGFP fluorescence, which was distributed all over the apical cell, labelling a structure that resembled the ER and surrounding the nucleus, suggesting that PINA was retained in the ER (Fig. 7A, right, asterisks). To quantify and confirm the intracellular distribution of PINA-EGFP, a region of interest (ROI) covering the tip of the apical cell was selected to obtain a fluorescence intensity histogram (Fig. 7B). In the apical cell expressing the full-length *CNIH2*, we observed a peak at lower values (low or no fluorescence), indicating that most of the ROI showed no fluorescence, associated with a limited localization of PINA at the cell apex; in contrast, under transformation with either, *CNIH2-141* or *CNIH2-137*, broader peaks were observed towards brighter pixels, signal associated with PINA-EGFP fluorescence, indicating a wider intracellular distribution of PINA within the ROI (Fig. 7B). Together, these results suggest that the putative phosphorylation and acidic domains of CNIH2 are important for interaction with the cargo and correct trafficking of the auxin transporter to the apical PM. Interestingly, the protonemal growth was altered in plants expressing the C-terminal truncations. In contrast to straight protonemata observed when WT *CNIH2* was expressed, expression of the truncations produced progressively curvier protonemata, with *CNIH2-141* producing wavy filaments (Fig. 7C, arrow) and *CNIH2-137* producing zigzagging filaments (Fig. 7C, arrow). It is likely that mislocalization of the PINA auxin transporter results in changes in the direction of auxin efflux, inducing an undulatory growth in protonemata. This effect is reminiscent of the rapid changes in polar localization of *Arabidopsis* PIN proteins in response to environmental or developmental cues such as embryonic development or root gravitropism (Kleine-Vehn et al., 2010).

## DISCUSSION

Here, we demonstrate that the polarized localization of PINA depends on its interaction with the cargo receptor CNIH2 as part of the secretory pathway (Figs 3, 4 and 5). We show that, among the two cornichon homologs present in *P. patens*, CNIH2 establishes a stronger interaction with PINA (Fig. 3A), an observation that correlates with PINA mislocalization in plants lacking CNIH2 but not of its paralog, CNIH1 (Fig. 3C; Fig. S7). Moreover, we demonstrate that the C terminus of CNIH2 appears to play an important role in its interaction with the cargo protein PINA (Fig. 6), as deletion of a domain containing putative phosphorylation sites (CNIH-141) leads to a stronger interaction and partial retention of the auxin transporter in the ER (Fig. 7A,B); in addition, removal of the acidic domain (CNIH-137) exacerbates this effect by completely preventing the apical localization of PINA in the apical cell of the protonemata, causing a clear retention of the transporter in the ER (Fig. 7A,B). Importantly, confirmation that the correct localization of PINA is dependent on its interaction with CNIH2 was obtained by the growth defects that were caused by the truncated versions of the cargo receptor, leading to undulating growth of protonemata when expressing either truncation (Fig. 7C).

A key point in the secretory pathway is the ER, in which the exit of membrane proteins is under the control of the COPII coat or directly by cargo receptors (Barlowe and Miller, 2013; Brandizzi and Barlowe, 2013). In this report, we have identified that moss cornichons are located at the ER and in puncta in protonemata cells (Fig. 4A; Fig. S9A); we demonstrated that the moss auxin efflux transporter PINA is a cargo protein for the moss cornichon cargo receptors, CNIH1 and CNIH2, interactions that occurred at the ER and in puncta throughout the cytoplasm (Fig. 3B), suggesting that moss cornichon proteins control the correct trafficking of PINA to the PM along the early secretory pathway. These observations agree with those reported for the cargo receptor Erv14 in yeast, which shows a predominant ER localization where it interacts with cargo membrane proteins for their recruitment to the ERES through its interaction with Sec24p and, eventually, within the COPII vesicles, before reaching the Golgi (Herzig et al., 2012; Pagant et al., 2015; Powers and Barlowe, 1998, 2002). Additional evidence supporting this conclusion is the higher affinity of PINA for CNIH2 (Fig. 3A), which could explain the alterations in early caulonemal development observed in the Δ*cnih2* mutant (Fig. 2A,D) as a result of PINA mislocalization (Fig. 3C). This trafficking defect can cause an alteration in export efficiency of auxin leading to its intracellular accumulation at the apical cell, as shown for the Δ*pinapinb* mutants, in which a reduced auxin export into the medium was reported (Thelander et al., 2017; Viaene et al., 2014), together with an accelerated differentiation of caulonemal cells, just as we observed for the Δ*cnih2* mutant (Fig. 2A,C). Confirmation that the correct localization of PINA is dependent on its interaction with CNIH2 was obtained by generating C-terminal truncations of the CNIH2 protein (Fig. 7A,B). This effect can be explained by the decrease in the strength of the protein-protein interaction that we observed between the truncated CNIH2-137 and PINA in the mbSUS assay (Fig. 6B), and agrees with previous reports demonstrating that the acidic domain in cornichon homologs from yeast and plants is important to establish a strong interaction with different cargo membrane proteins and necessary for their correct localization to the PM (Rosas-Santiago et al., 2017). Moreover, and this may be particular for CNIH2, the presence of a potential phosphorylation residue (T148) appears to play an important role in the interaction with PINA, as evinced by the mislocalization of the transporter caused by removal of the T/S domain and the associated morphological changes caused in the protonema (Fig. 7C). This result opens the possibility that the phosphorylated receptor could play a positive role in regulating cargo release to the PM. We observed abnormal positioning of branching cells in the cornichon mutants (Fig. 2A,B; Fig. S5). Until now, it has only been reported that mutants in Myosin VIII (Wu et al., 2011) and a Vapyrin-like (VPY-like) protein (Rathgeb et al., 2020) exhibit these branching defects. In moss protonemata, one of these cornichon-interacting proteins could be a VPY-like protein according to its localization to puncta in the cytoplasm and around the nucleus, coincident to that we observed for CNIH2 (Fig. 5A). The localization of VPY and VPY-like proteins was denoted as vapyrin bodies related to the TGN and endosomes, but also associated with the ER (Bapaume et al., 2019; Feddermann et al., 2010; Liu et al., 2019; Pumplin et al., 2010; Rathgeb et al., 2020). Although the function of Vapyrin (VPY) and VPY-like proteins is unknown, they are only found in plants and possess a vesicle-associated protein (VAP) domain at their N terminus, and several ankyrin repeat domains at the C terminus, with both domains predicted to be involved in protein-protein interactions (Feddermann et al., 2010; Pumplin et al., 2010), opening the possibility that they could associate with CNIH2.

According to the interaction between CNIH1 and CNIH2 with PINA that we demonstrate in this work, it is possible that the phenotypes observed in the *cnih1*, Δ*cnih2* and *cnih1*Δ*cnih2* gametophores (Fig. 2E,F) might result from an alteration in the intracellular concentration of auxins. Supporting this view are the bigger gametophores reported for the Δ*pinb* mutant (Bennett et al., 2014), which has a phenotype similar to that we observed for the Δ*cnih2* mutant, confirming the role of CNIH2 as the cargo receptor for PINA and suggesting that PINB could also depend on a similar interaction for its incorporation into the PM.

The independence of PINA from CNIH1 to reach its apical location (Fig. 3C), despite having observed the interaction of the two proteins (Fig. 3A,B), together with the pleiotropic effects generated by the single cornichon mutants (Fig. 2), indicate that they may be caused by the mistargeting of additional membrane proteins as cornichon receptors interact and mediate the trafficking of many membrane proteins that pass through the secretory route (Herzig et al., 2012; Rosas-Santiago et al., 2015), including those related to cell proliferation and expansion, processes that are required for the formation of the gametophore shoot (Kawade et al., 2020).

The observation that CNIH2 is associated with Sec23G (Fig. 5), one of the seven isoforms of Sec23 (Chang et al., 2022), leads us to propose that CNIH2 is located to a Sec23G structured ERES, as part of the pathway that controls PINA exit from the ER (Herzig et al., 2012; Pagant et al., 2015; Rosas-Santiago et al., 2017).

Our results demonstrate that the PIN transporters travel along the secretory pathway to reach their target, with the assistance of the cargo receptor cornichon as an element necessary for the correct selection of the cargo. This mechanism, together with other processes, such as endocytosis, recycling and/or transcytosis, or the participation of the retromer complex and/or the exocyst (Doyle et al., 2015; Drdová et al., 2013; Geldner et al., 2003; Jaillais et al., 2007), tightly regulate the required amount of PIN transporter to control plant development, expanding our knowledge on the participation of different trafficking mechanisms for PIN proteins.

## MATERIALS AND METHODS

### Protein sequence analysis

Protein sequence alignments and phylogenetic analyses were obtained with the CLC Main Workbench v6 software (Qiagen). A phylogenetic tree was used from matrix pairwise data and, using UPGMA algorithm, we performed 100 bootstrap rounds. For the prediction of serine, threonine and/or tyrosine protein phosphorylation sites in *Pp*CNIH1 and *Pp*CNIH2 proteins, we employed the server NetPhos3.1 (https://services.healthtech.dtu.dk/services/NetPhos-3.1/) (Blom et al., 1999).

### Moss strains, growth conditions and protoplast isolation

*Physcomitrium patens* (‘Gransden’ WT strain) was used in this study. Protonema was propagated routinely by spreading the tissue onto a cellophane disk laid inside a Petri dish with PpNH_4_ medium [1.03 mM MgSO_4_, 1.86 mM KH_2_PO_4_, 3.3 mM Ca(NO)_3_, 45 mM FeSO_4_, 2.72 mM (NH_4_)_2_-tartrate, 9.93 mM H_3_BO_4_, 220 nM CuSO_4_, 1.966 mM MnCl_2_, 231 nM CoCl_2_, 191 nM ZnSO_4_, 169 nM KI and 103 nM Na_2_MoO_4_] supplemented with 0.7% agar; the moss was grown at 24°C under 70 µE m^−2^ s^−1^ light and 16 h light/8 h dark regime. The YFP-Golgi (YFP-*Gm*Man) line was provided by Dr Magdalena Bezanilla (Dartmouth College, MA, USA), *PpPINA_pro_:PpPINA-EGFP* line (referred to as PINA-EGFP) was provided by Dr Sundberg (Swedish University of Agricultural Science, Uppsala, Sweden).

For protoplast isolation, 7-day-old protonema tissue was digested in a 0.5% driselase (Sigma-Aldrich) and 8.5% mannitol (Sigma-Aldrich) solution for 1 h in a shaker at room temperature. Protoplasts were passed through one layer of Miracloth (Merck-Millipore) to remove undigested tissue and centrifuged at 1600 rpm (250 ***g***) for 7 min and washed in 8.5% mannitol solution, three times. The protoplasts were resuspended in top agar (PpNH_4_ medium, 6% mannitol, 10 mM CaCl_2_, 0.3% agar) or in liquid plating media (PpNH_4_ medium with 8.5% mannitol, 10 mM CaCl_2_). Finally, for protonema regeneration, protoplasts were transferred into PRMB medium (PpNH_4_ medium supplemented with 6% mannitol, 10 mM CaCl_2_ and 0.7% agar). We employed the protoplasts for moss transformation and as starting material for morphological evaluation.

### Plasmids

Almost all the entry and expression plasmids employed in this work were obtained from Dr Magdalena Bezanilla and are compatible with the Gateway technology (Invitrogen). Plasmids for transient expression were: pDONR221 and pTZeoUBI-gate. Plasmids for generating gene mutation using the CRISPR-Cas9 system were: pENTR-PpU6P-L1L5r, L5L4, L4L3 and L3L2 and the pMZeo-Cas9-gate. For the generation of knock-in constructs by CRISPR-Cas9 & HDR, the plasmids used were: pENTR-PpU6P-L1L2, pDONR-P1P4, pDONR-P3P2, pDONR-R4R3-3XmRuby-C, pDONR-R4R3-3XmNeon-C, pMZeo-Cas9-gate, pMH-Cas9-PpU6P-sgRNA-sec23g, pDONR-B1-Sec23G 5′ arm-B4, pDONR-B3-Sec23G 3′ arm-B2 and pGEM-gate (https://www.addgene.org/Magdalena_Bezanilla/). The plasmids employed to generate knockout mutants by the homologous recombination technique were pDONR-P1P4, pDONR-P3P2, pDONR-R4R3-loxP-Hygro-loxP and the pGEM-gate.

### Protospacers constructs for *CNIH1* CRISPR-Cas9 mutagenesis

To generate the *Cnih1* protospacers construct, the CRISPR-Cas9 system was employed, as previously described (Mallett et al., 2019). A protospacer was designed using the CRISPOR online software (crispor.tefor.net) using *P. patens* (Phytozome V11) as the genome and *Streptococcus pyogenes* (5′NGG3′) as the protospacer adjacent motif (PAM) parameters. Four protospacers were chosen based on high specificity scores and low off-target frequency along the *CNIH1* gene. Each protospacer was designed to target one of the four predicted exons of the *CNIH1* gene. Each protospacer and its reverse complement were synthesized as oligonucleotides, adding the CCAT- sequence at the 5′ end of each to create sticky ends compatible with *BsaI* (Thermo Fisher Scientific). Each pair of oligonucleotides was annealed by PCR (500 pmol of each, 10 µl total volume, with the following setting conditions: 98°C 3 min, 0.1°C/s to oligo Tm, hold 10 min, 0.1°C/s to 25°C). In parallel, the pENTR-PpU6P-L1L5r, L5L4, L4L3 and L3L2 entry vectors were linearized with *BsaI* for 14-16 h at 37°C. Each protospacer was ligated with their respective entry vectors, using the Instant Sticky-end Ligation Master Mix (New England Biolabs), following the manufacturer's specifications, and generating pENTR-L1L5r-protospacer 1, pENTR-L5L4-protospacer 2, pENTR-L4L3-protospacer 3 and pENTR-L3L2-protospacer 4 constructs. The correct sequence of these entry constructs was confirmed by sequencing and finally recombined into the pMZeo-Cas9-gate expression vector by a four-fragment multisite Gateway recombination reaction (Invitrogen) with the LR clonase II plus, following the manufacturer's specifications. The pMZeo-Cas9/protospacers plasmid construct was transformed into moss protoplasts using the polyethylene glycol-mediated (PEG) transformation protocol (Mallet et al., 2019).

### *CNIH2-3XmRuby* and *SEC23G-3XmNeon* tagging by CRISPR-Cas9 & HDR

To generate the *CNIH2-3XmRuby* transgenic moss line, we employed the CRISPR-Cas9 system together with the HDR system described in Mallett et al. (2019). To generate the pMZeo-Cas9/cn2 tag protospacer plasmid construct, first, a protospacer was designed and chosen with the parameters as described above. In this case the protospacer was chosen closest to the stop codon of the *CNIH2* gene. Then, the selected protospacer and its reverse complement were synthesized as oligonucleotides, adding the CCAT- sequence at the 5′ end of each, to create sticky ends compatible with *BsaI-*linearized entry vectors. The oligonucleotides were annealed by PCR and cloned into the pENTR-PpU6P-L1L2 entry vector as previously described; this construct was sent for sequencing and, finally, it was recombined into the pMZeo-Cas9-gate expression vector by an LR clonase reaction following manufacturer's specifications (Invitrogen).

To generate homology arms for tagging *CNIH2*, we amplified two fragments of 1109 bp upstream of the start codon and 1101 bp downstream of the *CNIH2* stop codon. The upstream and downstream fragments were cloned in the pDONR-P1P4 and pDONR-P3P2 vectors, respectively, using a BP clonase reaction. To generate the HDR construct required for *CNIH2-3XmRuby* C-terminal gene tag, we used the three fragment Multisite Gateway cloning system (Invitrogen) and recombined the pDONR-B1-*CNIH2* 5′ arm-B4, pDONR-R4R3-3XmRuby-C, pDONR-B3-C*NIH2* 3′ arm-B2 into the pGEM-gate destination vector by a triple LR reaction. The pMZeo-Cas9/*CNIH2* and pGEM-*CNIH2-3XmRuby* constructs were co-transformed into WT moss protoplasts.

To generate the *SEC23G-3XmNeon* transgenic moss line, the pMH-Cas9-PpU6P-sgRNA-*SEC23G* construct was used as a protospacer plasmid. To produce the HDR construct required for construction of the *SEC23G-3XmNeon* C-terminal gene tag, the pDONR-B1-*SEC23G*23 5′ arm-B4, pDONR-B3-*SEC23G* 3′ arm-B2 and pDONR-R4R3-3XmNeon-C constructs were used and recombined into the pGEM-gate destination vector by a triple LR reaction. pMH-Cas9-PpU6P-sgRNA-*SEC23G* and pGEM-*SEC23G-3XmNeon* constructs were finally co-transformed in protoplasts using the *CNIH2-3XmRuby* line as background.

### Generation of *PpCNIH2* knockout constructs by homologous recombination

To obtain the Δ*cnih2* null mutant, a replacement construct was generated; a 1196 bp PCR-amplicon upstream from the 5′ start site of the gene and a 1284 bp PCR-amplicon downstream from the stop codon were amplified from genomic DNA and cloned independently by a BP clonase reaction into the pDONR-P1P4 and pDONR-P3P2 plasmids, respectively, according to the manufacturer's specifications (Invitrogen). The primers contained the appropriate *attb* sites. A *PmeI* restriction site was designed and inserted at both 5′ and 3′ ends to linearize the knockout construct. The entry clones were corroborated by sequencing. To generate the homologous DNA donor template flanking the hygromycin resistance cassette, the entry clones with homologous arms and the pDONR-R4R3-loxP-Hygro-loxP plasmid were recombined into the pGEM-gate plasmid in a three-fragment multisite Gateway recombination (Invitrogen) using an LR II clonase plus reaction. Finally, a *PmeI* digestion of the homologous DNA donor template was performed and the linearized construct was precipitated with ethanol before moss protoplast transformation.

For generation of the cornichon double mutant, the parental line Δ*cnih1-23* was employed to create the Δ*cnih2* null mutant by homologous recombination, in which the *CNIH2* gene was replaced by the hygromycin resistance cassette, as described above.

### Moss transformation and selection of transformants

For protoplast moss transformation, the PEG transformation protocol was used (Mallet et al., 2019). For homologous recombination replacement, protoplasts were transformed with 15 or 30 μg of DNA. For the CRISPR-Cas9 mutagenesis, protoplasts were transformed with 15 μg of total DNA construct. For the CRISPR-Cas9 & HDR system protocol, protoplasts were co-transformed with 7.5 μg total of CRISPR-Cas9/protospacer plasmid construct and 7.5 μg of total homology plasmid. After transformation, plants were allowed to regenerate on PRMB medium for 4 days.

To select stable transformants of the Δ*cnih2* null mutants, plants were moved to PpNH_4_ medium containing hygromycin (15 μg/ml). The potential knockout transformants were cycled on and off in antibiotic plates for two 1-week intervals. For the selection of the CRISPR-Cas9 and the CRISPR-Cas9 & HDR transgenic lines, Zeocin (50 μg/ml) was employed. For the selection of the *SEC23G-3XmNeon* line, the transformants were grown on hygromycin-containing medium. After plants were under selection for 1 week, the cellophane was changed to fresh PpNH_4_ medium three times every week. Finally, the surviving plants were picked with sterile tweezers and grown on PpNH_4_ medium (without cellophane) for 3-4 weeks to allow maximal growth for genomic DNA extraction.

### Genetic analyses

To identify and select the colonies transformed with the *CNIH2-3XmRuby* or *SEC23G-3XmNeon* constructs, internal primers 1463-Fw and 1462-Rv or s23G-Int-F and S23g-Int-R (Table S1) were employed, respectively. For the genotyping of potential Δ*cnih1* single and double cornichon null mutants, primers were designed that allowed the amplification of 1000 bp flanking the mutation target site of single guide RNAs (sgRNAs) to observe the differences in PCR band size in comparison with the WT DNA region. Potential mutant moss colonies were identified and screened using the primers 1268-Fw and 1512-Rv (Table S1), and those in which the amplified PCR product was different from the WT size were selected. The genomic DNA of two potential single and double mutant lines was isolated and confirmation of the PCR product was obtained by sequencing. *CNIH1* gene mutant expression analysis was corroborated by PCR amplification in the Δ*cnih1* single and double mutant using the primers 252-Fw and 253-Rv (Table S1).

For genotyping the potential Δ*cnih2* null mutant line in single and double mutants, a PCR amplification was performed to synthesize the expected fragments of 5331 bp for *CNIH2* genomic DNA and 6035 bp for the knockout (hygromycin cassette) genomic DNA, using the primers 327-Fw and 328-Rv (Table S1). Confirmation of the replacement of the *CNIH2* gene by the hygromycin cassette was obtained by PCR analyses. In one reaction, using the primers 327-Fw and 319-Rv (Table S1), a 3059 bp PCR fragment corresponding to the 5′ homologous genomic region and the hygromycin cassette sequence was obtained, as expected. By using the primers 320-Fw and 328-Rv (Table S1), a second 4120 bp PCR fragment corresponding to the 3′ homologous genomic region and the hygromycin cassette sequence was also obtained, as anticipated. Absence of the *CNIH2* transcript in the Δ*cnih2* single and double mutants was confirmed by PCR amplification using the primers 298-Fw and 299-Rv (Table S1).

### DNA extraction by the CTAB method

For genomic DNA extraction, the cetyl trimethylammonium bromide (CTAB) method was employed, with the following modifications. A DNA extraction buffer (0.1 M Tris-HCl pH 8.0, 1.4 M NaCl, 2.0% CTAB, 20 mM Na_2_EDTA, 2.0% PVP-40) was previously prepared, to which we added β-mercaptoethanol (10 mM) and ascorbic acid (0.1%). After that, half of a moss colony was taken with sterile tweezers and, to remove excess water, the tissue was squeezed between several sheets of sterile filter paper (Whatman 3 M). The dried tissue was collected in a 1.5 ml sterile tube and immediately frozen in liquid nitrogen. The tissue was homogenized in a prechilled microcentrifuge pestle, adding 200 µl of DNA extraction buffer previously pre-warmed to 65°C and ground until a green liquid mix was formed. Then, 1 µl RNAse A (10 mg/ml) was added, and the tubes were incubated at 65°C for 5 min. After that, 100 µl of the mix chloroform:isoamyl alcohol (24:1) was added to the tubes and mixed thoroughly; the phases were separated by centrifugation at 12,000 rpm (13306 ***g***) for 10 min. The upper phase was collected to precipitate the DNA by adding an equal volume of isopropanol and mixed by vortex; followed by incubation at −20°C for at least 15 min. Working in a sterile hood, the supernatant was discarded, and the pellet was washed with 70% ethanol and air dried. Finally, the pellet was dissolved in 25 µl sterile ddH_2_O.

### RNA extraction and cDNA synthesis

For extraction of total RNA from protonema tissue to genotype the cornichon single and double mutants, Plant RNA purification Reagent (Invitrogen) was used, followed by DNAse I treatment (Thermo Fisher Scientific) according to the manufacturer's recommendations. For the analysis of cornichon gene expression, synthesis of cDNA was performed using an oligo(dT) primer and RevertAid M-MuLV reverse transcriptase (Thermo Fisher Scientific) following the manufacturer's protocol.

### Morphological analysis

For protonema morphological analysis, protoplasts obtained from 7-day-old protonemal tissue were resuspended in 0.5 μl liquid plating medium (PpNH_4_ medium supplemented with 8.5% mannitol and 10 mM CaCl_2_) and plated in PRMB medium plates (PpNH_4_ medium supplemented with 6% mannitol, 10 mM CaCl_2_ and 0.7% Agar) with cellophane overlays for 4 days, and then transferred to PpNH_4_ medium for 4 days. After this, the protonema tissue was mounted onto a microscope slide with PpNH_4_ liquid medium and covered with a cover slide. Tissue was observed under an inverted microscope (Nikon, Eclipse) and images were acquired using a digital camera (Nikon 7500). Calcofluor was used to enhance cell morphology by staining the cell wall and observations were made in a fluorescence stereomicroscope (Zeiss Axioscope) with an objective of 10×; images were obtained with a CCD camera (Photometrics CoolSnapcf Monochromatic) and analyzed with ImageJ/Fiji (Schindelin et al., 2012). Images from every line were adjusted to sharpen and improve the cell division lines. The caulonema/cloronema ratio was manually quantified.

Gametophore morphological analysis was performed in a colony assay. Seven-day-old protonema tissue sections of 3×3 mm were placed carefully with forceps on PpNH_4_ solid medium and were grown under 24°C 16 h light/8 h dark photoperiod for 4 weeks. The gametophores with more than five fillids and well-formed rhizoids (under stereomicroscope observation) were collected and counted. The moss colony and gametophore images were taken with a digital camera (Olympus or Sony α).

### Protonema sample preparation for BFA treatment

For BFA (Sigma-Aldrich) treatment, several pieces of cellophane containing a 1-week-old protonema were carefully cut with a scalpel, placing them on PpNH_4_ medium plates supplemented with 50 µM of BFA or DMSO (control plates). Plates were left in incubation for 24 h at 24°C (16 h light /8 h dark regime). For confocal microscopy observation of protonema tissue, a piece of cellophane containing the protonema was cut and placed face down onto an agar pad containing 50 µl of Hoagland's medium [4 mM KNO_3_, 2 mM KH_2_PO_4_, 1 mM Ca(NO_3_)_2_, 89 µM Fe citrate, 300 µM MgSO_4_, 9.93 µM H_3_BO_3_, 220 nM CuSO_4_, 1.966 µM MnCl_2_, 231 nM CoCl_2_, 191 nM ZnSO_4_, 169 nM KI, 103 nM Na_2_MoO_4_] with 1% agar and 1% sucrose. The cellophane was removed by sliding it carefully, leaving the tissue attached to the agar pad. Then, 10 μl of liquid Hoagland's medium and 1% sucrose were added onto the tissue, covered with a coverslip, and sealed with 1:1:1 Vaseline:Lanolin:Paraffin mixture. To maintain the tissue under exposure to BFA during observation, 50 µM BFA was added to the mix of liquid Hoagland's medium.

### Confocal microscopy and co-localization assay

For *CNIH2-3XmRuby*, fluorescence was observed using a confocal microscope (LSC Nikon Ti2), employing an excitation wavelength of 561 nm and an emission of 647 nm with a 1.49 NA, Apo 60× immersion oil objective. The fluorescence of the YFP-Golgi marker was obtained by exciting at 488 nm (0.5% laser power) and observing at 524 nm, with a 60× oil immersion objective with a 1.3 NA using an inverted confocal microscope (Olympus FV1000). For both transgenic lines, optical sections of 0.75 to 1.00 µm in the *z*-axis were taken for acquisition of images. The images were viewed with ImageJ (Schindelin et al., 2012).

For co-localization analysis between SEC23G and CNIH2, an inverted confocal microscope (Olympus FV1000) was employed. Fluorescence from *SEC23G-3XmNeon* was obtained by exciting at 488 nm (0.5% power laser) and observed at an emission of 510 nm. *CNIH2-3XmRuby* fluorescence was excited at 543 nm (5% power laser) and observed at 655 nm emission in a 60× oil immersion objective with a 1.3 NA. Confocal images were taken every 0.75 to 1.00 µm in the *z*-axis. The images were viewed and analyzed in ImageJ. For co-localization analysis, the Co-loc2 plugin in ImageJ was employed to derive a Pearson's correlation coefficient; values above 0.5 are indicative of colocalization.

Dot size quantification was made in ImageJ by sharpening and thresholding three different optical confocal images from *CNIH-3XmRuby*, *SEC23G-3XmNeon* and YFP-Golgi lines. Vesicles were selected with the wand tool and the area of 35 random vesicles was measured and plotted.

### Mating-based split ubiquitin yeast system

For the detection of protein-protein interactions, we employed mbSUS in yeast cells (Lalonde et al., 2010; Obrdlik et al., 2004). Plasmid constructs were generated using the coding sequence for *PINA*, *CNIH1*, *CNIH2*, *CNIH2-141* and *CNIH2-137* without the stop codon and cloned into the pDONR221 plasmid by a BP reaction (Invitrogen). Once we verified by sequencing the correct cloned genes, we used an LR clonase reaction (Invitrogen) to transfer the *PINA* gene to the pMETYC_GW (Cub clones) and *CNIH1*, *CNIH2*, *CNIH2-141* and *CNIH2-137* to the pXN32_GW (Nub clones) vectors. For the mbSUS assay, yeast media were prepared as previously described in Jones et al. (2014) and Lalonde et al. (2010). The THY.AP4 (*MATa ura3, leu2, lexA::LacZ::trp1 lexA::HIS3 lexA::ADE2*) and THY.AP5 (*MATα URA3, leu2, trp1, his3 loxP::ade2*) yeast strains were transformed with the pMETYC_GW and pNX32_GW vectors, respectively. Yeast cells were transformed using the LiAc treatment (Gietz and Woods, 2006).

### BiFC assay in leaf epidermal cells from *N. benthamiana*

Complementation of EYFP fluorescence (BiFC) experiments were carried out according to Rosas-Santiago et al. (2015). To obtain the expression clone for each gene employed in the BiFC assays, an LR gateway-based recombination reaction (Invitrogen) with either pYFC43 or pYFN43 was carried out. Leaves were infiltrated with the constructs *pYFC43-PpPINA*, *pYFC43-AtPIP2A*, *pYFN43-PpCNIH1*, *pYFN43-PpCNIH2* and *pYFN43-AtPIP2A*. *Agrobacterium tumefaciens* GV3101 strain cells were transformed with each construct by electroporation and grown in 30 ml of LB medium with rifampicin (50 μg ml^–1^) and spectinomycin (50 μg ml^–1^) or kanamycin (50 μg ml^–1^) at 28°C at an OD_600_ of 0.3 to 0.5. Leaves were infiltrated with a bacterial culture with an OD_600_ of 0.3 resuspended in sodium phosphate buffer (pH 7.0), 0.1 mM acetosyringone (Sigma-Aldrich) and 28 mM glucose.

### Changes in subcellular localization of PINA-EGFP in moss protonema and complementation assays

To observe changes in PINA subcellular location in cornichon single mutants, we employed the reporter line *PpPINApro:PpPINA-EFGP*. The Δ*cnih1* single mutant was obtained using the CRISPR-Cas9 technique, whereas the Δ*cnih2* single mutant was obtained by homologous recombination (see above). Selected single cornichon mutants were picked and observed under an Olympus FV1000 confocal microscope to identify the location of the PINA-EFGP fluorescence in protonema apical cells. A piece of cellophane containing 7-day-old protonema was cut and placed onto a microscope slide containing 20 µl of PpNH_4_ liquid medium and covered with a coverslip and sealed with nail polish for observation of EGFP (excitation and emission wavelengths of 488 nm and 510 nm, respectively, with 0.5% laser power in an inverted confocal microscopy (Olympus FV1000).

To clone the *CNIH2* WT gene and the C-terminal truncated versions (*CNIH2-141* and *CNIH2-137*), total RNA was obtained from *P. patens* protonemal tissue using the Plant RNA Purification Reagent (Invitrogen), followed by a DNAse I treatment (Thermo Fisher Scientific) following the manufacturer's recommendations. Synthesis of cDNA was performed using an oligo(dT) primer and RevertAid M-MuLV reverse transcriptase (Thermo Fisher Scientific) following the manufacturer's protocol. The predicted coding sequences for *CNIH2*, *CNIH2-141* and *CNIH2-137* were amplified by PCR with the appropriate *attb* sites (B1 and B2) and each PCR product was cloned in the pDONR-221 entry vector by a BP clonase reaction. These constructs then were cloned in pMZeo-Ubi-gate expression plasmid using an LR reaction. The entry and the expression clones were verified by restriction enzyme analysis and by sequencing at the sequencing unit of Instituto de Biotecnología, UNAM, México.

For complementation assays, each construct was transformed into protoplasts for transient expression in the moss single mutant Δ*cnih2-3*/*PpPINA-EFGP*. After transformation, plants were allowed to regenerate on PRMB medium for 4 days and then were maintained in zeocin selection for 12 days before being observed under a confocal microscope as described above. Each image corresponding to each complementation with CNIH2 and C-terminal truncated versions were captured via *z*-projection and changed to an 8-bit image to measure the intensity of PINA-EGFP fluorescence (0-255), after an area of 46.8 µm^2^ was delimited with a polygon ROI at the apex zone of the protonema cell.

### Statistical analysis

The data are presented as bar charts and box plots were obtained using Origin software (OriginLab). All statistical analysis was performed using Excel. A two-tailed Student's *t-*test for unpaired data with equal variance was used. For gametophore analysis, ANOVA and Tukey-Kramer post hoc test was performed. *P*-values >0.05 were reported as not significant (ns). *P-*values ≤0.05 or 0.001 were reported as significant and highly significant, respectively.

### Accession numbers

Plant and algae cornichon homolog sequences were obtained from ARAMEMNON (http://aramemnon.botanik.uni-koeln.de/), Phytozome (https://phytozome-next.jgi.doe.gov/) and EnsemblPlants for algae *Chara braunii* (https://plants.ensembl.org/Chara_braunii/Info/Index). Names and accession number are as follows: *Arabidopsis thaliana* (*At*CNIH1: *At*3g12180.1; *At*CNIH2: *At*1g12340.1; *At*CNIH3: *At*1g62880.1; *At*CNIH4: *At*1g12390.1; *At*CNIH5: *At*4g12090.1), *Oryza sativa* (*Os*CNIH1: *Os*06g-04500.1; *Os*CNIH2: *Os*12g32180.1), *Zea mays* (*Zm*CNIH1: GRMZM2G073023.01; *Zm*CNIH2: GRMZM2G018885.01; *Zm*CNIH3: GRMZM2G124658.01), *Populus trichocarpa* (*Pt*CNIH4: *Potri*001g116100.1; *Pt*CNIH3: *Potri*003g116400.1; *Pt*CNIH1: *Potri*006g057300.3; *Pt*CNIH2: *Potri*016g051000.1), *Ananas comosus* (*Ac*CNIH: *Aco*015328.1), *Zostera marina* (*Zma*CNIH1: *Zosma*28g00840; *Zma*CNIH3: *Zosma42g*010-60; *Zma*CNIH2: *Zosma*153g00310), *Selaginella moellendorfi* [*Sm*CNIH: 93931(PAC:15402723)], *Physcomitrium patens* (*Pp*CNIH1: *Pp*3c11_17020V3.3; *Pp*CNIH2: *Pp*3c7_11500V3.3), *Marchantia polymorpha* (*Mp*CNIH: *Mapoly*0124s0019), *Chlamydomonas reinhardtii* (*Cr*CNIH: *Cr*e01.g036550_4532), *Dunaliella salina* (*Ds*CNIH: *Dusal*.0011s00016) and *Chara braunii* (*Cb*CNIH: GBG61058.1). Fungi cornichon homolog sequences were obtained from the Yeast Genome database (www.yeastgenome.org) and NCBI: *Saccharomyces cerevisiae* (*Sc*Erv14p: SGD:S000003022), *Schizo-saccharomyces pombe* (*Sp*CNIH: NP_594657.1), *Neurospora crassa* (*Nc*CNIH: XP_011395262.1) and *Aspergillus nidulans* (*An*CNIH: XP_662799.1).

## Supplementary Material

10.1242/develop.201635_sup1 Supplementary information
