# Supplementary material for: A cornichon protein controls polar localization of the PINA auxin transporter in Physcomitrium patens
Source: Development. 2023 May 5;150(9):dev201635. doi: 10.1242/dev.201635 (PMC10259512; doi:10.1242/dev.201635)
Supplement: Supplementary information [file develop-150-201635-s1.pdf]

Figure S1

A

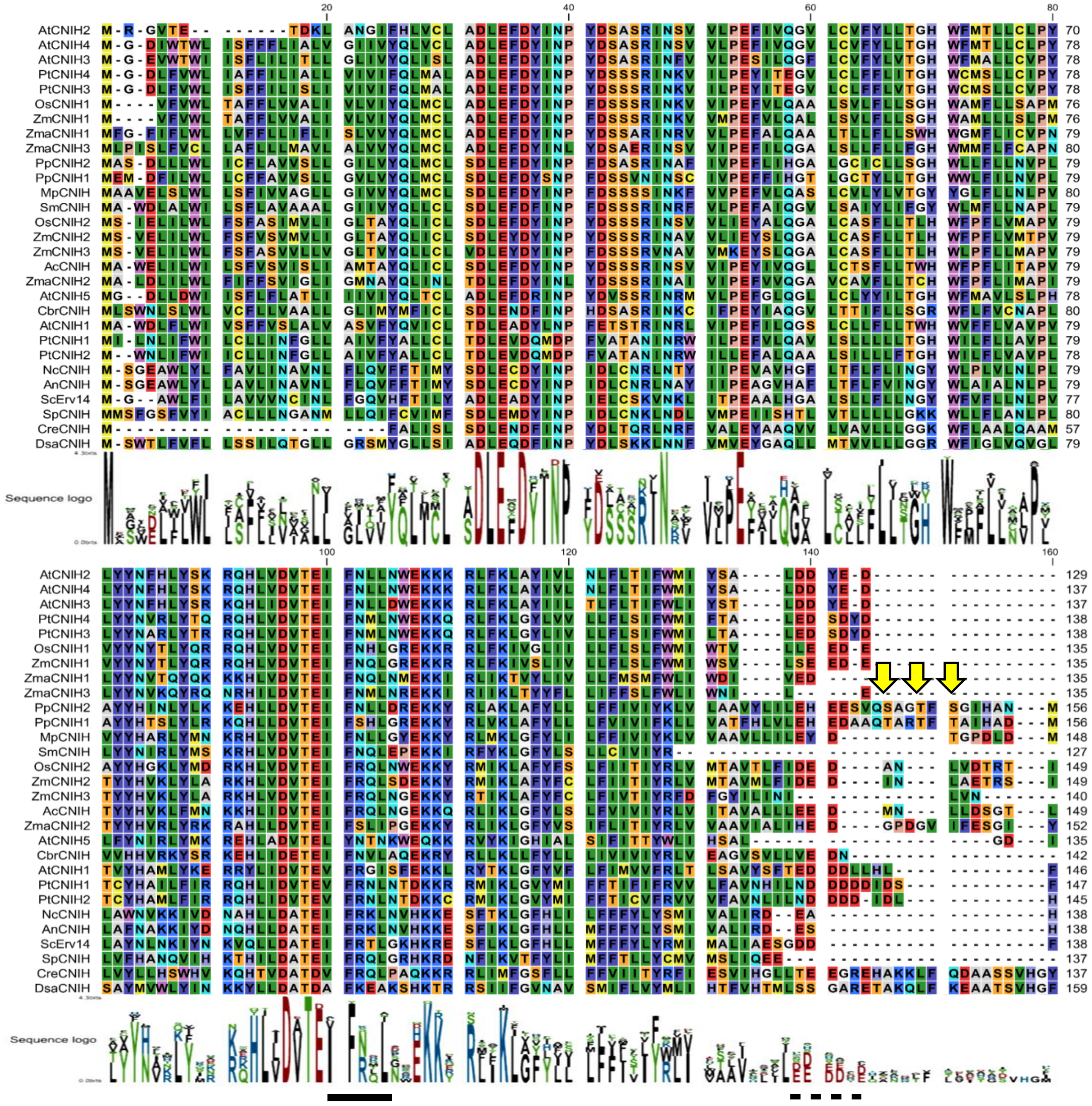

Development • Supplementary information

Fig. S1. Multiple amino acid sequence alignment of cornichon homolog proteins and putative phosphorylation sites in moss homologs. Amino acid sequence alignment of cornichon homologs from algae, plants, and fungi; solid and dashed bars show the consensus motif IFNXL, the acidic motif (Ac. Dom), respectively. Arrows indicate predicted phosphorylation sites on Ser and/or Thr residues identified by the NetPhos3.1 prediction server.

Development • Supplementary information

Figure S2

A

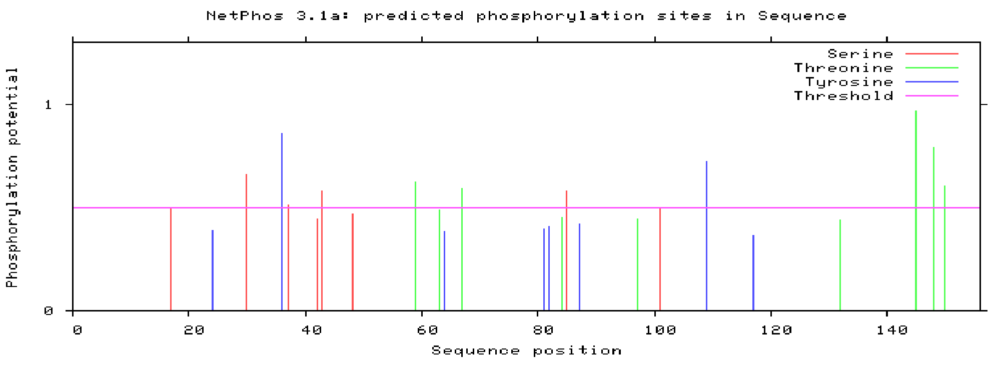

B

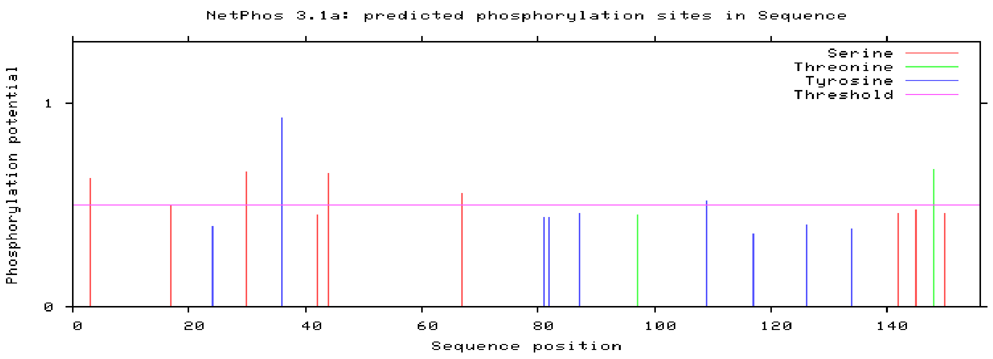

Fig. S2. In silico analysis of putative phosphorylation sites of moss cornichon proteins and proteins pairwise comparison matrix. Predicted phosphorylation sites for CNIH1 A) and CNIH2 B) proteins. Serine, Threonine and Tyrosine are shown in red, green and blue, respectively; in silico analysis was performed with the NetPhos3.1 server (https://services.healthtech.dtu.dk/service.php?NetPhos-3.1).

Development • Supplementary information

Figure S3

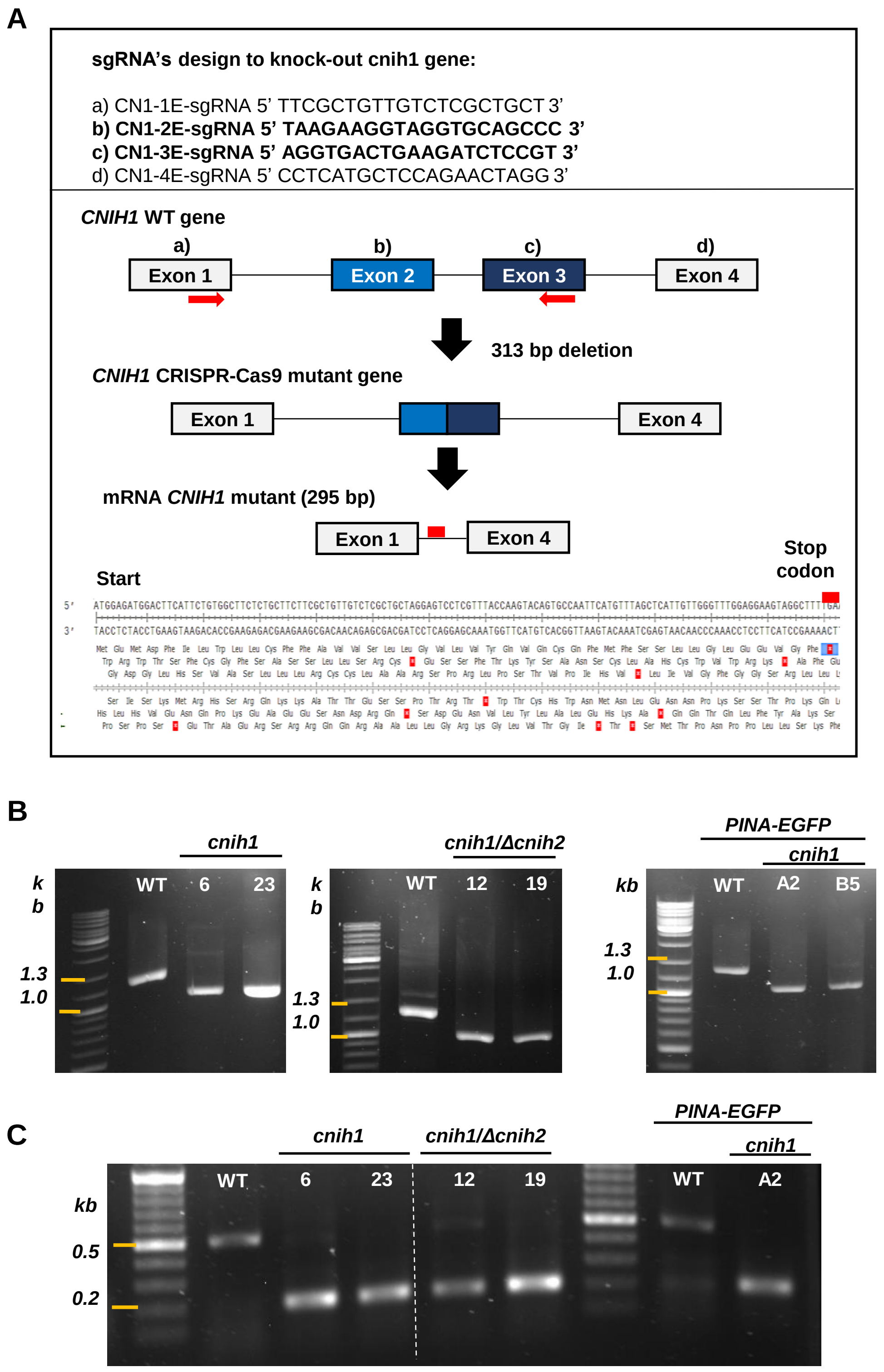

Development • Supplementary information

Fig. S3. Generation of *cnih1* mutant lines by the CRISPR-Cas9 system. A) Schematic strategy for mutation of the *CNIH1* gene by the CRISPR-Cas9 system. Four different sgRNA were synthetized (a to d); each sgRNA targeted one of the four exons of the *CNIH1* gene. Only the b and c sgRNA's (bold type) were efficient and deleted a total of 313 bp (removing the second intron and part of Exon 2 and Exon 3) resulting in the *cnih1* mutant line. This mutation generated an in-frame premature stop codon at nucleotide 132 (indicated by a red line) that codifies for 43 amino acids (predicted peptide size of 5 kDa). **B)** Comparison of the PCR products from the WT *CNIH1* gene (1,309 bp); the single *cnih1* mutant lines (1,000 bp) (#6 and #23) and double *cnih1/Δcnih2* mutant lines (#12, #19) in WT parental line, and in the reporter PINA-EGFP lines (A2, B5). Amplified PCR bands from genomic DNA extractions. **C)** Comparison between WT *CNIH1* and *cnih1* single mutant coding sequences (cDNA). Agarose DNA gel (1%) shows amplified PCR bands from cDNA of WT *CNIH1* (468 bp); *cnih1* single mutant lines (295 bp) (#6 and #23) and double *cnih1/Δcnih2* mutant lines (#12 and #19), and in the *cnih1* PINA-EGFP single mutant line (A2).

Figure S4

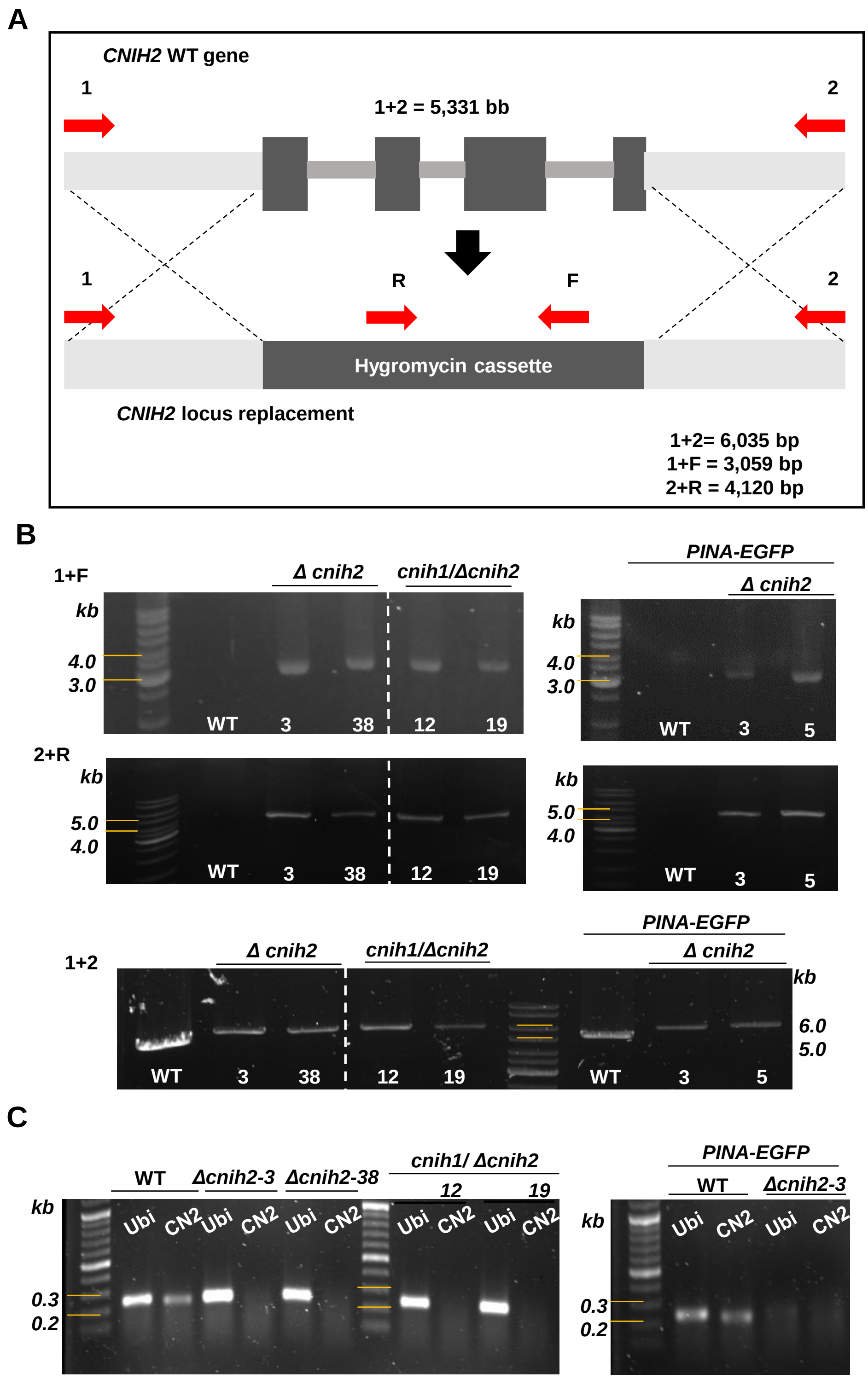

Development • Supplementary information

Fig. S4. Generation of *Δcnih2* mutant lines and their genotypification. A) *CNIH2* disruption strategy showing the genomic location of *CNIH2* locus and primers (red arrows) for genetic analyses. **B)** Agarose DNA gel (1%) showing the presence and the replacement by the hygromycin cassette by PCR amplified products from WT and *Δcnih2* single mutant lines (#3 and #38), *cnih1/Δcnih2* double mutants (#12 and #19), and *Δcnih2* single mutant lines (#3 and #5) in the PINA-EGFP genetic line. **C)** Agarose DNA gel (1%) shows amplified PCR bands from cDNA of *CNIH2* (468 bp) in WT and in PINA-GFP lines, in comparison with the absence of *CNIH2* mRNA transcript in the *Δcnih2* single mutant lines (#3 and #38), *cnih1/Δcnih2* double mutant lines (#12 and #19) and *Δcnih2* /PINA-GFP line (#3).

Development • Supplementary information

Figure S5

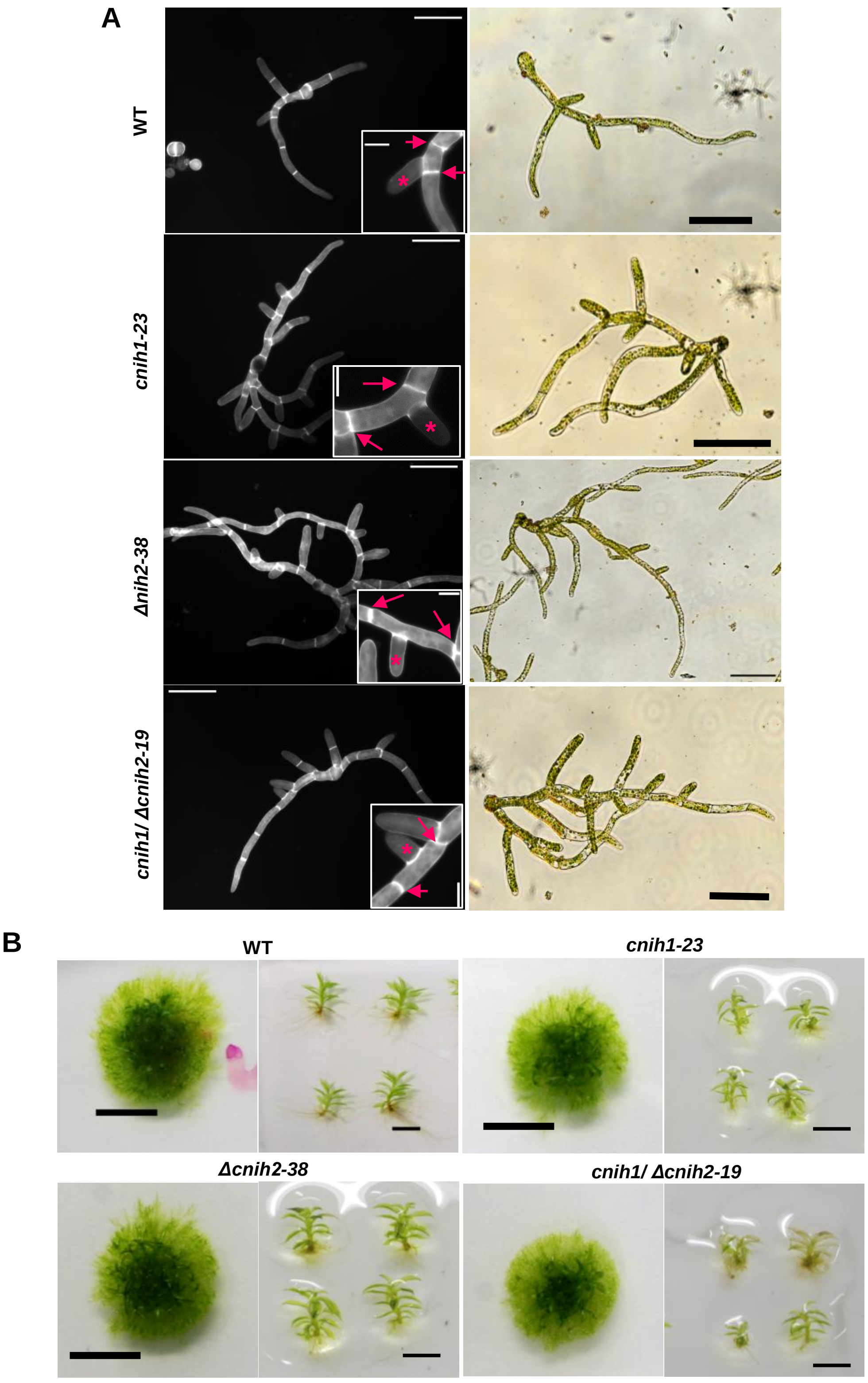
A
WT
cnih1-23
Δnih2-38
cnih1/ Δcnih2-19
B
WT
cnih1-23
Δcnih2-38
cnih1/ Δcnih2-19

Fig. S5. Cornichon mutants have pleiotropic effects during the moss life cycle. A) (Left panel) Protonema from WT and cornichon mutants stained with Calcofluor White after 7 d growth, visualized in an epifluorescence microscope; scale = 100 µm. Insets shows cell divisions (arrows) and lateral initial branch cell (*). (Right panel) Brightfield images of seven-day-old protonema from WT and cornichon mutants, Scale 100 µm. **B)** Colony (top, scale 5 mm) and individual gametophores (bottom, scale 2 mm) from WT and cornichon mutants after four weeks of growth.

Development • Supplementary information

Figure S6

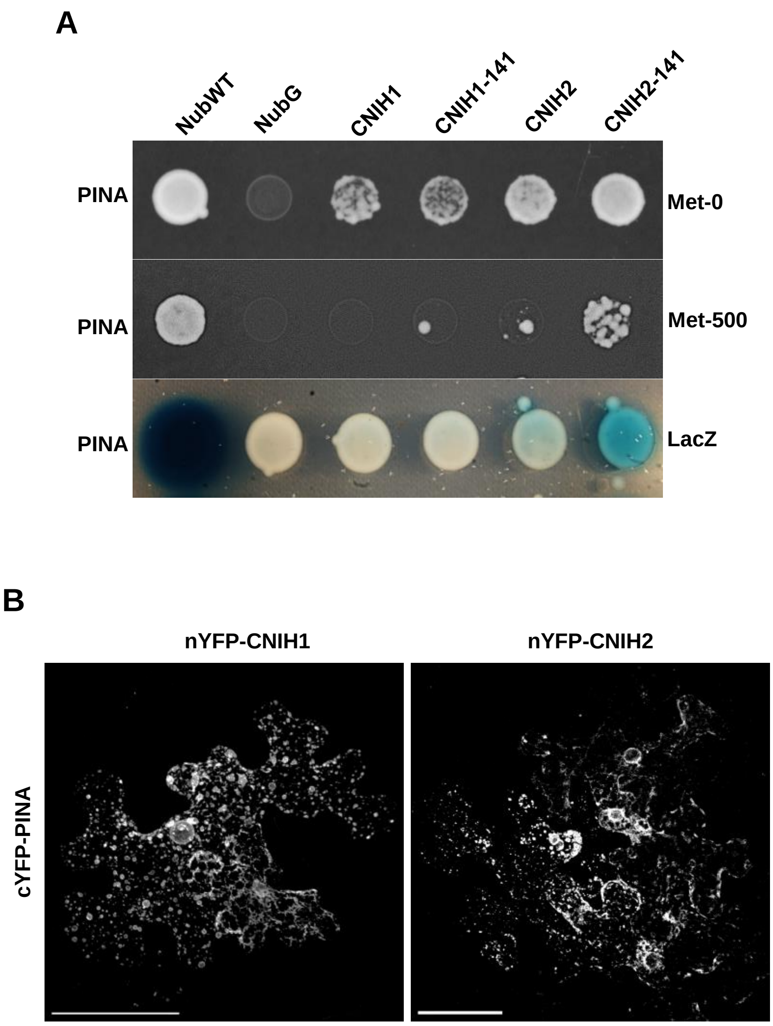

Fig. S6. CNIH2 protein is the cargo receptor for the auxin transporter PINA. A) Protein-protein interaction identified by the mbSUS assay with the moss cornichon WT, CNIH1-141 and CNIH2-141 proteins (Nub fusions) and the auxin transporter PINA (Cub fusion). Yeast cell growth in selection medium (Met-0); the strength of the interaction was confirmed by cell growth inhibition under repressive selection conditions (Met-500) and by the lower activity of LacZ (intensity of the bluish color). NubWT and NubG were used as false negative and false positive controls, respectively. **B)** Original images of Figure 3B, showing interaction between PINA and CNIH1 or CNIH2 was confirmed by reconstitution of split-YFP fluorescence by the co-expression of nYFP-CNIH's with c-YFP-PINA proteins, scale = 50 µm.

Development • Supplementary information

Figure S7

A

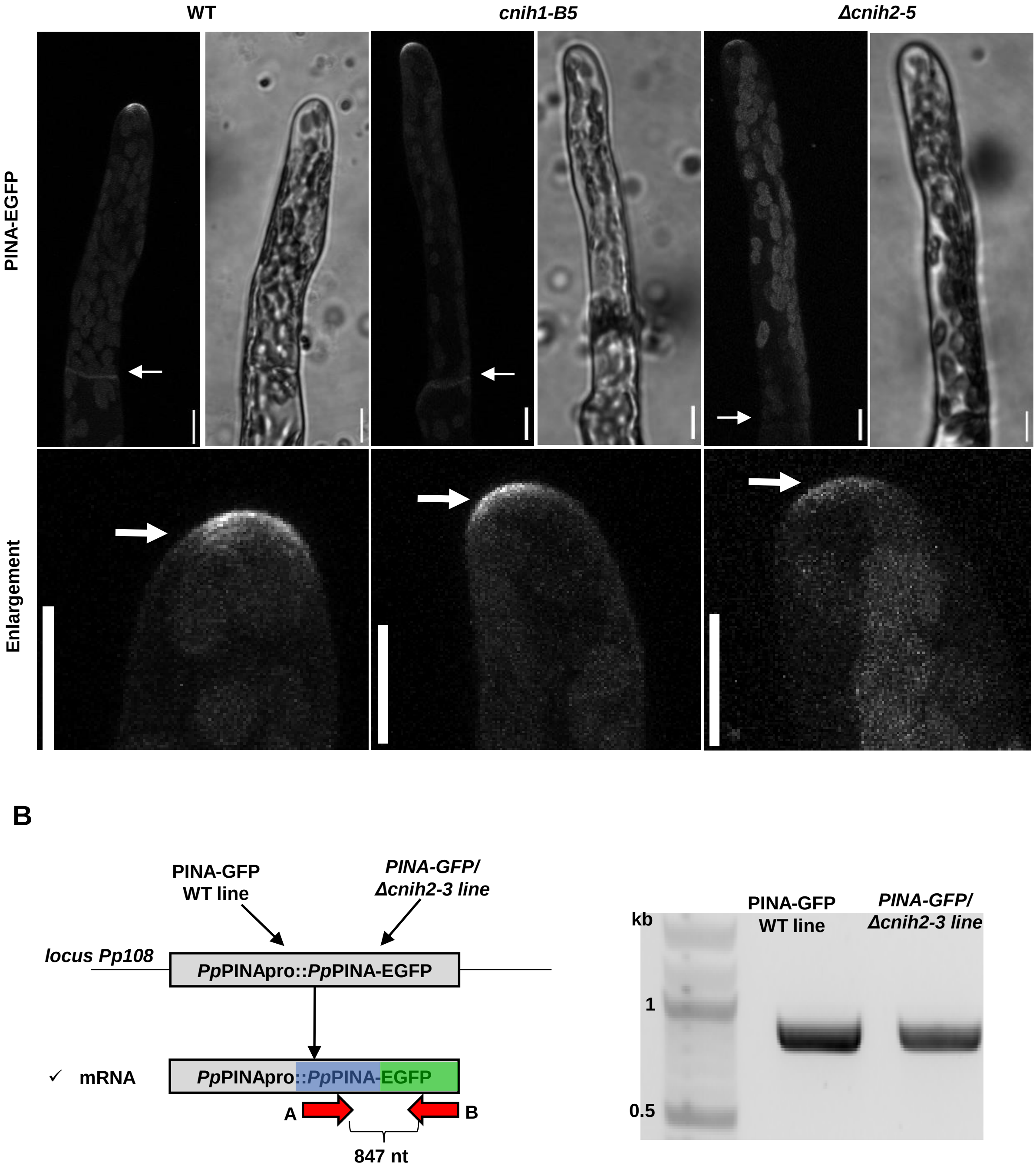

Development • Supplementary information

Fig. S7. Subcellular localization of the auxin efflux transporter PINA in additional *cnih1* and *Δcnih2* single mutants. A) (top) Localization of PINA in WT and cornichon single mutants in a protonema apical cell. Fluorescence at the tip and the base (arrow) was maintained in the *cnih1-B5* single mutant, but not in the *Δcnih2-5* single mutant. (bottom) ROI enlargement of PINA-EGFP fluorescence at the tip of the apical protonema cells from WT, *cnih1-B5*, and *Δcnih2-5* mutant lines. Scale = 10 µm. **B)** (left) Schematic representation of the *Pp*PINApro::*Pp*PINA-EGFP construct inserted at the locus *Pp108* in WT (PINA-EGFP) and *Δcnih2-3* single mutant line; primers A and B were used to amplify a 847 nt corresponding 130 nt of PINA coding sequence (primer A) and 717 nt of the EGFP coding sequence (primer B) in the PINA-EGFP reporter line parental/background; (right) 1% agarose DNA gel showing expected PCR products confirming the presence of the PINA-EGFP transcript in both, over-expressing and *Δcnih2-3* mutant lines.

Development • Supplementary information

Figure S8

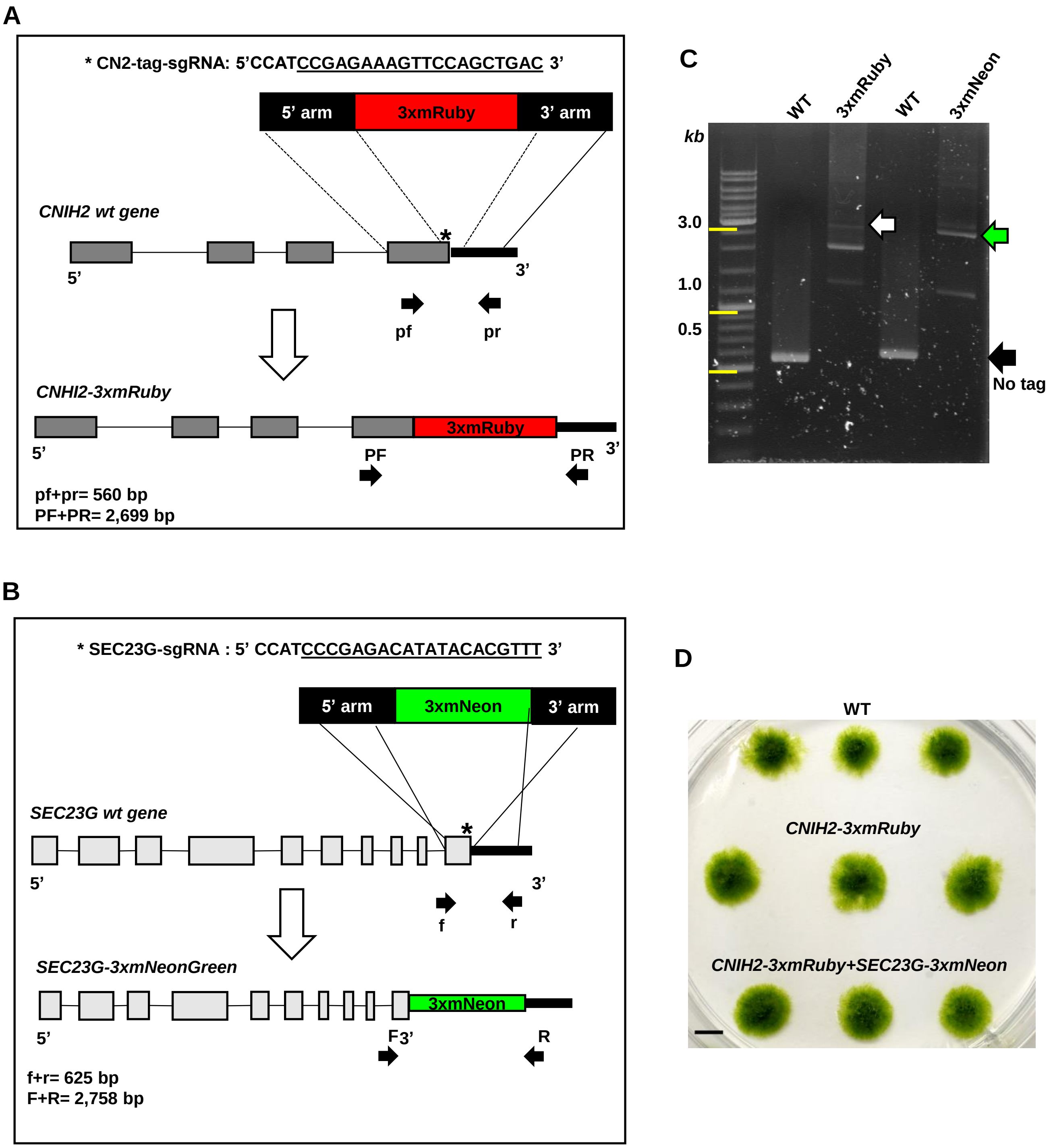

Development • Supplementary information

Fig. S8. Generation of CNIH2-3xmRuby and SEC23G-3xmNeon Knock-in lines by CRISPR-Cas9 & HDR. A) Design and representation of CNIH2-3xmRuby stable line at the C-terminus of the gene. sgRNA sequence guide for making a double break in DNA (asterisk) and 3xmRuby coding sequence flanked by recombination homolog sequences. Primers pf and pr amplified a fragment of 557 bp by PCR in WT line without inserting the tag. Primers PF and PR amplified a fragment of 2,699 bp by PCR in WT line with the insertion of a 3xmRuby tag. **B)** Design and representation of SEC23G-3xmNeon stable line at the C-terminus of the gene. sgRNA sequence guide for making a double break in DNA (asterisk) and 3xmNeon coding sequence flanked by recombination homolog sequences. Primers f and r amplified a fragment of 625 bp by PCR in the WT line without inserting the tag. Primers F and R amplified a fragment of 2,758 bp by PCR in WT line with the insertion of a 3xmNeon tag. **C)** 1% agarose DNA gel showing expected PCR products in the knock-in CNIH2-3xmRuby line (white arrow), SEC23G-3xmNeon line (green arrow), and without any tag (black arrow). **D)** Moss colonies from CNIH2-3mxRuby and CNIH2-3xmRuby+SEC23G-3xmNeon lines grown together with the WT line. Scale= 2 mm.

Figure S9

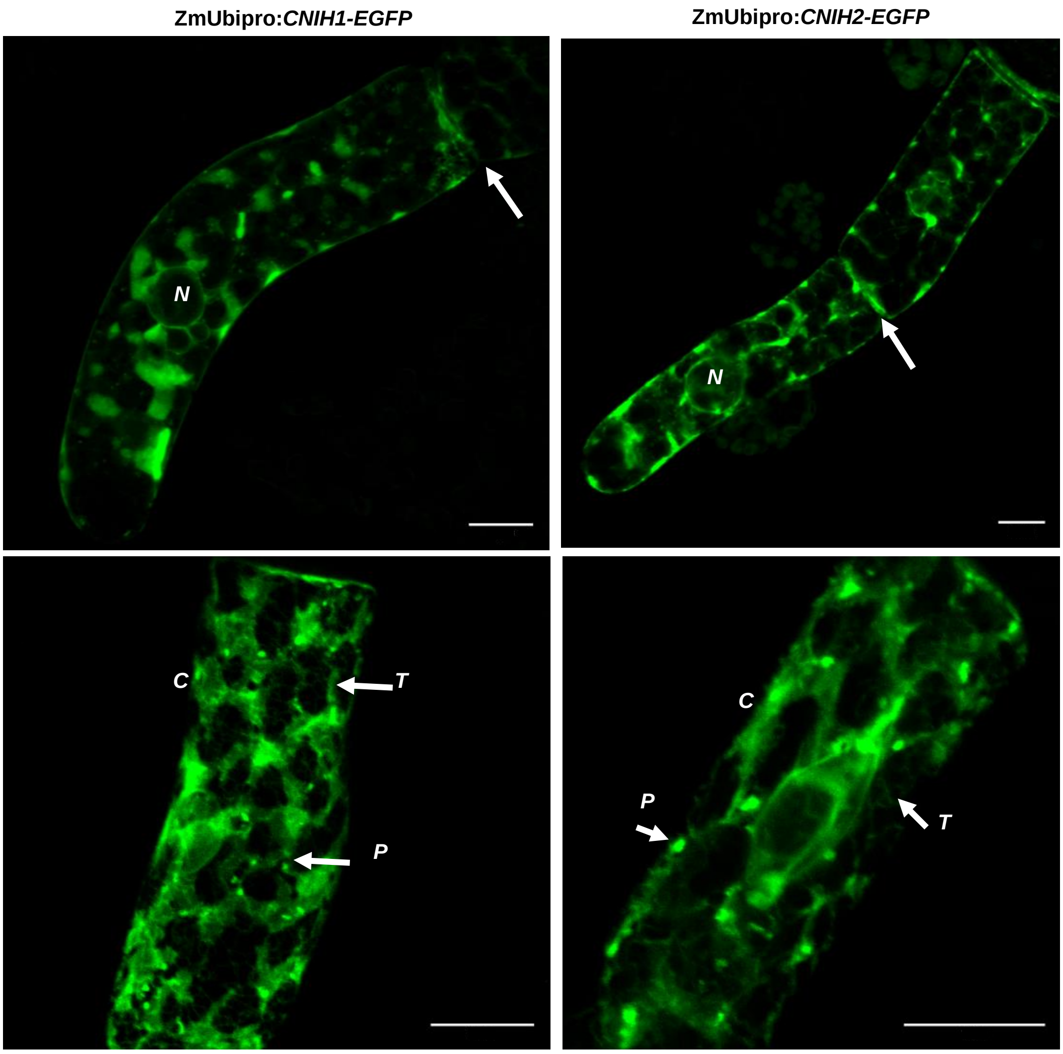

Fig. S9. Overexpression of moss cornichon proteins localized mainly at ER but also in puncta. Confocal images showing the subcellular localization of transiently expressed *ZmUbipro::CNIH1-EGFP* (left column) and *ZmUbipro:CNIH2-EGFP* (right column) from seven-day-old apical protonemal cells. Arrows indicate localization of the cell plate for both proteins (top row). N = nucleus. Identification of moss cornichons at ER subdomains as tubules (T) and cisternae (C), and in puncta below ER (P) (bottom row), scale 10 µm.

Development • Supplementary information

Figure S10

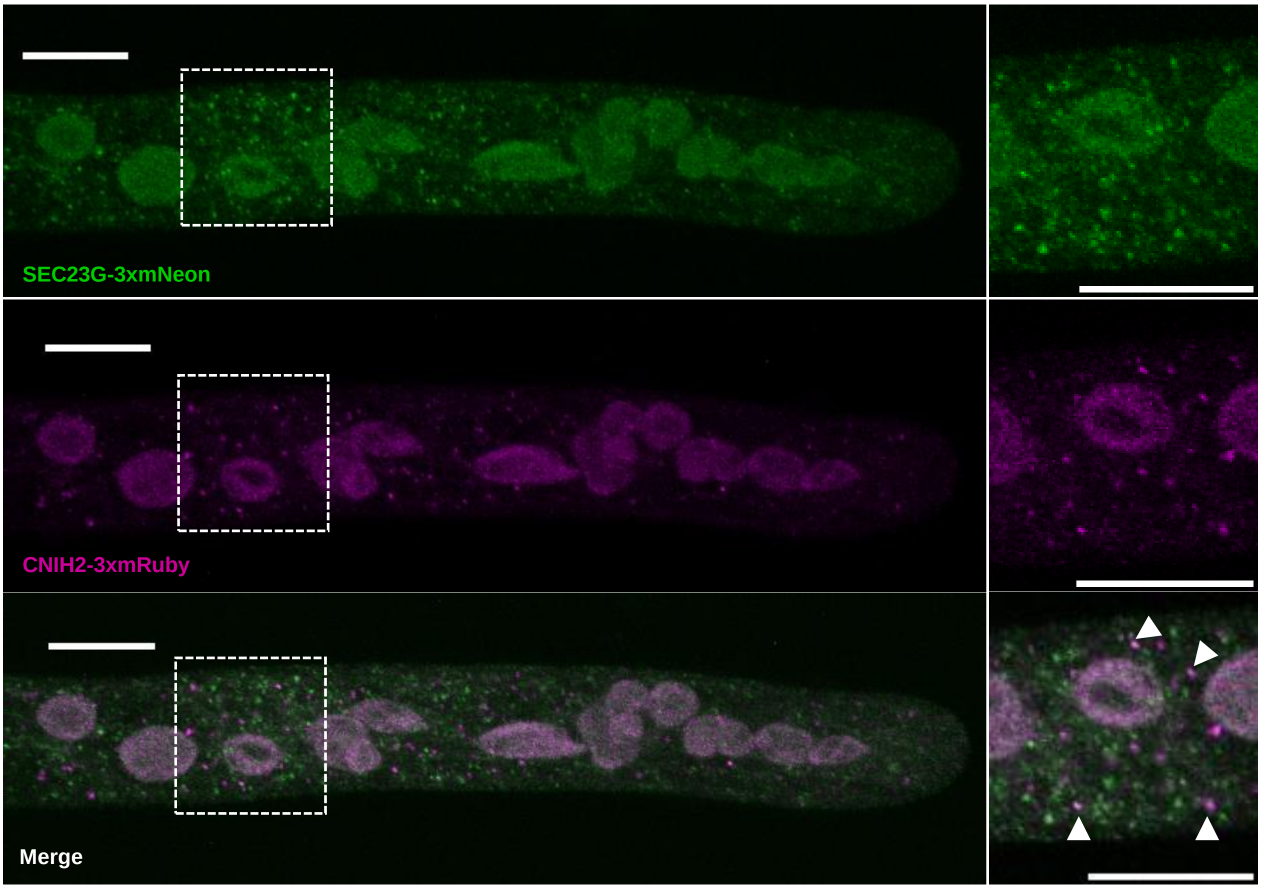

Fig. S10. Co-localization of CNIH2 and SEC23G. Original images of endogenous SEC23G (top panel), CNIH2 (middle panel) and merge images (bottom panel) of endogenous CNIH2 and SEC23G tagged proteins in a protonemal apical cell; (right) enlargement of the region delimited (dashed squares). Representative Z-projection with maximal intensity confocal image; scale 10 µm.

Figure S11

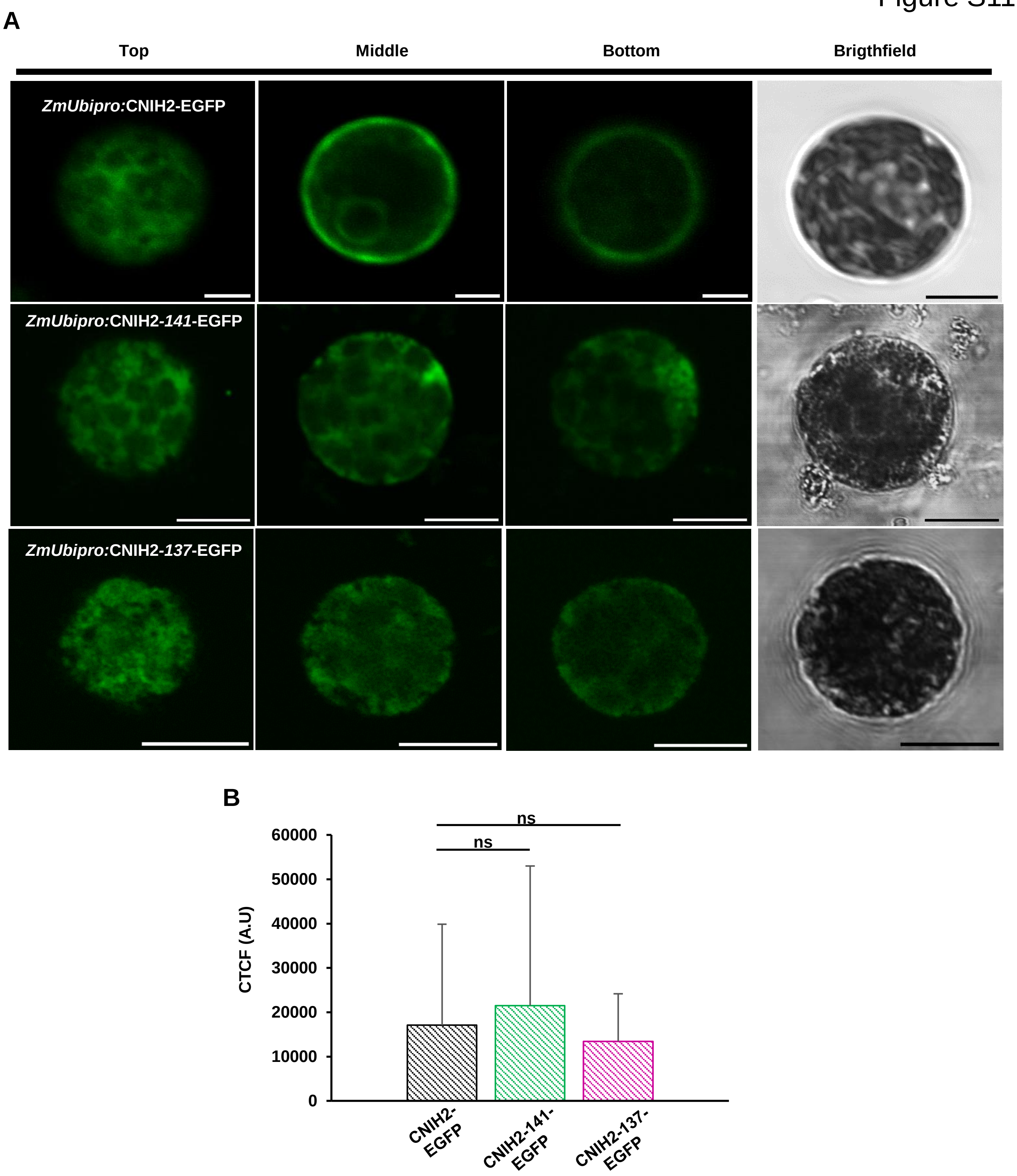
A
Top
Middle
Bottom
Brigthfield
ZmUbipro:CNIH2-EGFP
ZmUbipro:CNIH2-141-EGFP
ZmUbipro:CNIH2-137-EGFP
B
CTCF (A.U)
60000
50000
40000
30000
20000
10000
0
ns
ns
CNIH2-EGFP
CNIH2-141-EGFP
CNIH2-137-EGFP

Fig. S11. Expression of WT CNIH2 and C-terminal truncated proteins in WT moss protoplasts. A) Confocal images showing localization of transiently overexpressed WT *ZmUbipro::CNIH2-EGFP* (top row) and C-terminus truncated protein versions *ZmUbipro::CNIH2-141-EGFP* (middle row) and *ZmUbipro::CNIH2-137-EGFP* (bottom row) in moss protoplasts at 48 h after transformation, showing a reticulate subcellular localization. Images from three individual optical sections (Top, Middle and Bottom) of the protoplasts. Scale 10 µm. **B)** Fluorescence intensity of moss protoplasts transformed with the WT and CNIH2 C-terminus truncated fusion constructs. CTFC = corrected total cell fluorescence. n = 4; data are the mean ± SD *t*-test was performed for statistics (ns $p \geq 0.05$).

Table S1. Primers list

Name	SEQUENCE 5' – 3'	References
Cloning		
attB1 (5' half sequence)	GGGGACAAGTTTGTACAAAAAAGCAGGCT	This study
attB2 (5' half sequence)	GGGGACCACTTTGTACAAGAAAGCTGGGT	This study
PpCNIH1-For	GTACAAAAAAGCAGGCTTCATGGAGATGGACTTC	This study
PpCNIH1-Rev	GTACAAGAAAGCTGGGTCCATGTCTGCGTGGATTG	This study
PpCNIH2-For	GTACAAAAAAGCAGGCTTCATGGCTTCCGATCTCC	This study
PpCNIH2-Rev	GTACAAGAAAGCTGGGTCCATGTTTGCGTGGATC	This study
PpCNIH2-141-Rev	GTACAAGAAAGCTGGGTCTTCCTCATGCTCAAGAATTAAG	This study
PpCNIH2-137-Rev	GTACAAGAAAGCTGGGTCAAGAATTAAGTAGACGGCTGC	This study
PpCNIH1 disruption by CRISPR-Cas9 system		
sgRNACN1-1E-null-Rv	AAACAGCAGCGAGACAACAGCGAA	
sgRNACN1-1E-null-Fwd	CCATTTCGCTGTTGTCTCGCTGCT	
sgRNACN1-2E-Fwd	CCATTAAGAAGGTAGGTGCAGCCC	This study
sgRNACN1-2E-Rv	AAACGGGCTGCACCTACCTTCTTA	This study
sgRNACN1-3E-Fwd	CCATACGGAGATCTTCAGTCACCT	This study
sgRNACN1-3E-Rv	AAACAGGTGACTGAAGATCTCCGT	This study
sgRNACN1 4E-Fwd	CCATCCTCATGCTCCAGAACTAGG	This study
sgRNACN1-4E-Rv	AAACCCTAGTTCTGGAGCATGAGG	This study
Genotyping PpCNIH1 CRISPR-Cas9 mutants		
CN1-null-Fwd	CCATTTCGCTGTTGTCTCGCTGCT	This study
D-cni1-KO-Rv	GGACGTATGGACTGAATCC	This study
PpCNIH2 Knock-out disruption by Homologous recombination		
ATTB1-CNI2-M-Fw	GGGGACAAGTTTGTACAAAAAAGCAGGCTGGGTTTAAACGATAGTGAGAGTGAGATGATTGAGG	This study
ATTB4-CNI2-M-Rv	GGGGACAACTTTGTATAGAAAAGTTGGGTGGCTCCCGGCTTTCGCTGCTCCTCTC	This study
ATTB3-CNI2-4R-Fw	GGGGACAACTTTGTATAATAAAGTTGTAATTCTCTTTGGTTCGTAGCCCATTGG	This study
ATTB2-CNI2-4R-Rv	GGGGACCACTTTGTACAAGAAAGCTGGGTAGTTTAAACAATTCATCTTCGCTTGAACTAC	This study
Genotyping PpCNIH2 knock-outs		
Higro-F	GTCTGTCGAGAAGTTTCTGATCG	
Higro-R	CGTCGGTTTCCACTATCGG	
CN2-outer-up-F	GGAACGTACATGAGATGTGTCAAG	This study
CN2-outer-DW-R	CTCCCTCGTGTACTCCTTCC	This study
C2-F	GCTGTTCTTGCTCAACGTTCC	This study
C2-R	CTGACTGGACAGATTCCTCATGC	This study
Ubi10-F	ACTACCCTGAAGTTGTATAGTTCGG	
UbI10-R	CAAGTCACATTACTTCGCTGTCTAG	
PpCNIH2 Knock-in generation by CRISPR-Cas9&HDR		
CN2-tag-Fwd	CCATCCGAGAAAGTTCCAGCTGAC	This study
CN2-tag-Rv	AAACGTCAGCTGGAACTTTCTCGG	This study
pENT-CN2-Up-mut-Fw	GAATCTGTaCAGTCAGCTGGAAC	This study
pENT-CN2-Up-mut-Rv	GCTGACTGtACAGATTCCTC	This study
B1-CNIH2-tag-Fwd	GGGGACAAGTTTGTACAAAAAAGCAGGCTCGTCCTTGCGTCTATCACATCACG	This study
B4-CNIH2-tag-Rv	GGGGACAACTTTGTATAGAAAAGTTGGGTGCATGTTTGCGTGGATCCCCGAGAAAG	This study
B3-CNIH2-tag-Fwd	GGGGACAACTTTGTATAATAAAGTTGCGTCCTTGTGACTGTCACACTGAACC	This study
B2-CNIH2-tag-Rv	GGGGACCACTTTGTACAAGAAAGCTGGGTAGCAAGACATGAGCTAGATACCAAC	This study
PpCNIH2-3xmRuby line screening		
cn2-downarm-Fwd	CATGTATCGTTTCTGTCATG	This study
cn2-uparm-Rv	GATGTCATGTCAATACCAATG	This study
PpSEC23G-3xmNeon line screening		
S23g-int-F	GCTACTGATCAATGTTGACTGG	This study
S23g-int-R	GAACTAGTTCACTACTCCACG	This study
Transcript expression of PpPINA-EGFP knock-in line		
EGFP-Fwd	TAAACGGCCACAAGTTCAGCG	This study
pinA-Rv	GAGAGGTGCCACCTATTTGCAACC	This study

Development • Supplementary information
