# Supplementary material for: A cornichon protein controls polar localization of the PINA auxin transporter in Physcomitrium patens
Source: Development. 2023 May 5;150(9):dev201635. doi: 10.1242/dev.201635 (PMC10259512; doi:10.1242/dev.201635)
Supplement: Supplementary file 1 [file develop-150-201635_review_history.pdf]

A cornichon protein controls polar localization of the PINA auxin transporter in Physcomitrium patens

Carolina Yanez-Dominguez, Daniel Lagunas-Gomez, Diana Milena Torres-Cifuentes, Magdalena Bezanilla and Omar Pantoja

DOI: 10.1242/dev.201635

Editor: Dominique Bergmann

Review timeline

Original submission:	23 January 2023
Editorial decision:	21 February 2023
First revision received:	28 March 2023
Accepted:	3 April 2023

Original submission

First decision letter

MS ID#: DEVELOP/2023/201635

MS TITLE: The role of cornichon proteins in the development of Physcomitrium patens and polar localization of the PINA auxin transporter

AUTHORS: Carolina Yanez-Dominguez, Daniel Lagunas-Gomez, Diana Milena Torres-Cifuentes, Magdalena Bezanilla, and Omar Pantoja

I have now received all the referees' reports on the above manuscript, and have reached a decision. The referees' comments are appended below, or you can access them online: please go to BenchPress and click on the 'Manuscripts with Decisions' queue in the Author Area.

As you will see, the referees express considerable interest in your work, but have some significant criticisms and recommend a substantial revision of your manuscript before we can consider publication. In particular there are concerns about the experimental design and controls for the BIFC experiments and concerns about whether some localization of proteins to subcellular domains may be artifactual due to damaged cells. Overall the Figure image quality was low. This may have been due to compression when creating the PDF for initial review, so this may be resolved when figure files are created following journal guidelines as described in source files below.

If you are able to revise the manuscript along the lines suggested, which may involve further experiments, I will be happy receive a revised version of the manuscript. Your revised paper will be re-reviewed by one or more of the original referees, and acceptance of your manuscript will depend on your addressing satisfactorily the reviewers' major concerns. Please also note that Development will normally permit only one round of major revision. If it would be helpful, you are welcome to contact us to discuss your revision in greater detail. Please send us a point-by-point response indicating your plans for addressing the referee’s comments, and we will look over this and provide further guidance.

Please attend to all of the reviewers' comments and ensure that you clearly highlight all changes made in the revised manuscript. Please avoid using 'Tracked changes' in Word files as these are lost in PDF conversion. I should be grateful if you would also provide a point-by-point response detailing

© 2023. Published by The Company of Biologists under the terms of the Creative Commons Attribution License (https://creativecommons.org/licenses/by/4.0/).

how you have dealt with the points raised by the reviewers in the 'Response to Reviewers' box. If you do not agree with any of their criticisms or suggestions please explain clearly why this is so.

Reviewer 1

Advance summary and potential significance to field

A fundamental question in cell biology remains understanding the specificity inherent to membrane trafficking. Often, researchers start with a cargo and ask how it arrives to a specific subcellular location. The work of Yanez-Dominguez et al flips this question and explores the function of a cargo receptor that is currently understudied in plants- CORNICHON-HOMOLOGUE1,2 (CNIH1,2). The authors are able to present compelling evidence that the P. patens CNIH2 protein is likely a cargo receptor for the auxin efflux protein– PINA. While work with CNIHs in other plants has yielded interesting results, the work in Yanez-Dominguez et al makes the significant and novel connection between CNIHs and auxin transport, the latter of which is considered to be foundational to plant development. There are, however, some criticisms about the manuscript that I would like to see addressed before full publication.

Comments for the author

1) The authors use CRISPR/Cas9 to generate single and double mutants of the moss CNIHs. Some of the phenotypes of these mutants, as presented, are a bit confusing. For instance, Figure 2c, cnih1 has no branching phenotype, but cnih2 does. However, the double mutant is not significantly different. Something similar occurs in Figure 2D. Could the authors provide some explanation as to what they think could be occurring? The authors focus the bulk of the article focusing on how CNIH2 effects PINA-EGFP localization. Is PINA-EGFP localization different in the single vs double mutants?

2) Is there any way to provide statistics for Figure 2B. The different between WT and the double mutants seems significant, but the single mutants are less convincing. Perhaps if presented as a measurement from the base of the cell to the middle of the occurring branch? In the double mutants, such data sets would have a lower average because of the increased frequency of mid-cell branches.

3) The authors claim that the cnih2 line undergoes caulonemal differentiation earlier than the WT and cnih1 mutant (but not the cnih1,2 mutant). They do this by reporting the ratio of caulonemata to chloronemata, which the authors write were quantified manually. How did they score these cell types? By cell wall angle? By length? I would like to see the authors use a more quantitative approach, here. These data are particularly important as they draw the connection between a cellular process (membrane targeting), and plant development (auxin transport/levels within the cell).

4) I am concerned by the fact that PINA-EGFP does not mislocalize in the cnih2 background, but rather disappears. In Supplemental Figure S5A, the authors show an additional cnih2 line with what looks to be rather normal, albeit faint, PINA-EGFP localization. If the model that the authors present is correct, then one would expect PINA-EGFP to accumulate in the ER in the cnih2 mutant (similar to what is seen in Figure 7A). Why does this not occur in the cnih2/PINA-EGFP line?

5) Authors should confirm with Development team regarding policies of active/passive voice, and gene nomenclature (cornichon homologue vs CORNICHON-HOMOLOGUE).

Reviewer 2

Advance summary and potential significance to field

This paper describes the role of cornichon proteins in the moss Physcomitrium patens. The authors suggest that PINA is a cargo protein of the cornichon ‘receptor’ CNIH2, and that CNIH2 is required for the polarization of PINA in filaments. The findings are certainly interesting and novel, and this research adds a nice new dimension to what is already known in the field.

© 2023. Published by The Company of Biologists under the terms of the Creative Commons Attribution License (https://creativecommons.org/licenses/by/4.0/).

Comments for the author

The following suggestions are being made to improve the manuscript (notably to improve the reader's understanding of the rationale behind the study, and to improve the quality of the figures shown):

Title – as it stands the title is really underselling this paper. Why not be bolder – this could simply be rephrased as 'Cornichon proteins are required for the polar localization of the PINA auxin transporter in Physcomitrium patens'.

Abstract – too much use of the word moss on line 35

Introduction – generally quite comprehensive but needs to be a little more accessible

- Line 42 – correct eucaryotic to eukaryotic

- Line 52 – correct 'has' to 'have'

- Line 80 – 'with no obvious phenotype' – presumably the authors are describing the respective mutants, but this is currently unclear.

- Line 84 – 'pairs of AtCNIHs' – context? Elaborate on what you mean by 'pairs'

- Line 99 – correct 'cycle' to 'cycled'

- Line 104 – vias?

- Line 107 – please expand on your description of the retromer complex – what is it?

- Line 111-113 – more explicitly needs to be explained

- Line 123 – the authors refer to PIN gene families, but this needs to be referenced – perhaps from Viaene et al., 2014 and/or Bennett et al., 2014?

- Line 132 (and consistently repeated thereafter) – CNIH's – remove apostrophe please

Results

- Lines 149 and 174 (and all other instances) – please do not refer to bryophytes as early diverging land plants – see commentary by McDaniel.

- Line 175 – please amend hepatic to liverwort; correct 'Sellaginella' to 'Selaginella'

- Lines 181-185 are written in an unclear manner – please rephrase

- Line 189 – this is the first time that the authors refer to the (delta)cnih2 mutant – please can you check/confirm the conventions for the use of this terminology? I know that it is the convention for a full gene deletion – is this also the case for a partial deletion?

- Line 200 - 'mutants exhibited abnormal branching' – this really isn't clear based on the figure shown. Is this the case, or do the authors indeed have better quality representative images?

- Figure S4 is completely superfluous
- Line 208 – '35-40% of the branching' – shown graphically but with no representative images

The double mutant has a 'corrected' phenotype, which is never really discussed throughout the manuscript – are CNIH1 and CNIH2 operating antagonistically? Is the cnih1 mutation dominant?

© 2023. Published by The Company of Biologists under the terms of the Creative Commons Attribution License (https://creativecommons.org/licenses/by/4.0/).

- MAJOR
- Lines 224-226 – the authors observe that the (delta)cnih2 mutant phenotype resembles that of the pinb mutants described elsewhere. Would it therefore not have made sense to perform these studies using the PINB reporter rather than the PINA reporter? Please can the authors comment on the phenotype of the pina mutants (if known)? Some more justification is required. Also, to add to this work, could PINB not have been included in some of the protein interaction studies described later in the manuscript? It seems that PINB is entirely neglected, and it is unclear why. Perhaps the reporter line isn't very good? Please do comment.

- Lines 239-242 – the ER localisation is assumed and not demonstrated – does the localisation match that of an ER marker line at the very least? I know that these are available.

- Line 246 – the authors flip flop between PIN transporters and auxin transporters – please be consistent.

- Line 248 – 'served as a negative control' – is this not simply a negative result?

- Supplementary Figure 5 is largely superfluous.

- Supplementary Figure 8 – these protoplasts do not look healthy at all, and thus nothing meaningful can be extracted from these images. Often punctate structures are formed in protoplasts – usually when they are about to explode… A nice spherical cell is required…

- Line 349 and Figure 7 – all unclear and needs an explanation

- Lines 363 and 385 and Figure 7C – these images are not striking and do not really highlight the phenotypes that the authors describe as highly prevalent

Methods

- Line 485 – amend 'proportionated' to 'provided'

- Line 486 – is Dra. correct?

- Line 608 – amend 'by' to 'for'

Figures (some addressed above)
- Figure 1: Please include the protein alignment in Supplementary data. This will enable the authors to enlarge the currently very unreadable phylogeny, which can only be read on maximum zoom on screen, and even then, the support values cannot be read. Do not refer to 'higher plants' – it is anyway unclear whether the authors are grouping all land plants as 'higher plants' relative to algae. The concerns with the claims made about this tree are that the support values are too low – either include more species to resolve the tree better (and thus increase the support values) or make claims less bold.

- Figure 2: The graphs, in their current form, are quite unreadable and look scruffy.

- Figure 3: The BiFC figures are barely visible. Furthermore, I am unconvinced that the authors have used the correct controls – have they considered some empty vector controls?

- Figure 4: Again, the figures are unclear. Can the authors use black and white for (A) and then use different colours for (B) to make the distinction between the two lines? In the figure legend, please correct 'rigth' to 'right'.

- Figure 5: Unless you squint, nothing is really visible in this figure. Please also recolour to green and magenta so that the merge is more meaningful. Please check scale bar in zoomed in version – surely the scale bar needs to be altered (or removed).

© 2023. Published by The Company of Biologists under the terms of the Creative Commons Attribution License (https://creativecommons.org/licenses/by/4.0/).

- Figure 7 – In (A), middle image doesn't appear diffuse at all, but right-hand image does. Can the authors colour GFP and autofluorescence differently? The graph in (B) doesn't match the representative images shown in (A). I have already highlighted issues with Figure 7C above.

- Figure S1 – Serine, Threonine, Tyrosine and Threshold are not aligned with their respective coloured lines – looks odd. Figure S1c is superfluous.
- Figure S3 – exon of CNIH2 WT gene needs to be brought forward.

- Figure S4 – earlier described as superfluous, especially if WT images aren't included alongside. I think it's OK to remove this entirely.

- Figure S5 – try to improve visibility of images; in (B) please either show or describe the location of the primers used. Does this not simply show that both WT and cnih2-3 contain the endogenous PINA gene? This (currently) does not demonstrate that the construct is contained within. Can PINA-EGFP be described as WT?
I suspect that auxin transport may be perturbed in some way in this line. Can the authors comment on this? I do think there is still merit in the results shown – clearly there is a difference between WT and the mutant lines.

- Figure S6 – please amend HR schematic. It is usually convention to show the construct recombining with the endogenous WT locus, and then the final recombined locus (i.e., put the construct at the top of each)

- Figure S8 – I have already commented on this above. The protoplasts are not of sufficient quality to make any bold claims. This looks like an artefact.

First revision

Author response to reviewers' comments

Reviewer 1 Comments for the Author:
1) The authors use CRISPR/Cas9 to generate single and double mutants of the moss CNIHs. Some of the phenotypes of these mutants, as presented, are a bit confusing. For instance, Figure 2c, cnih1 has no branching phenotype, but cnih2 does. However, the double mutant is not significantly different. Something similar occurs in Figure 2D. Could the authors provide some explanation as to what they think could be occurring? The authors focus the bulk of the article focusing on how CNIH2 effects PINA-EGFP localization. Is PINA-EGFP localization different in the single vs double mutants?

As Reviewer 2 comments, we believe that CNIH1 and CNIH2 seem to operate antagonistically with the phenotype of the double mutant dominated by the mutation of cnih1, according to the phenotypes shown in Fig. 2. We did not analyze the localization of PINA in the double mutant because of the stronger interaction we observed between CNIH2 and PINA with the mbSUS (Fig. 3A). Moreover, in the cnih1 mutant, where endogenous CNIH2 is present, PINA was localized correctly, but not in the cnih2 mutant, where even endogenous CNIH1 is present, which together with the weaker interaction between CNIH1 and PINA, suggest that PINA is not a cargo for CNIH1.

2) Is there any way to provide statistics for Figure 2B. The different between WT and the double mutants seems significant, but the single mutants are less convincing. Perhaps if presented as a measurement from the base of the cell to the middle of the occurring branch? In the double mutants, such data sets would have a lower average because of the increased frequency of mid-cell branches.

We have quantified more protonemas of each WT and mutant lines and changed Fig. 2B showing the results as percentage of anormal branch cells per plant, where it can be seen that in the single mutant cnih1 and in the double mutant anormal branching is more frequent in

© 2023. Published by The Company of Biologists under the terms of the Creative Commons Attribution License (https://creativecommons.org/licenses/by/4.0/).

comparison with the WT line; this conclusion is supported by the statistical analysis of Student's t test for paired data with equal variance was used.

3) The authors claim that the cnih2 line undergoes caulonemal differentiation earlier than the WT and cnih1 mutant (but not the cnih1,2 mutant). They do this by reporting the ratio of caulonemata to chloronemata, which the authors write were quantified manually. How did they score these cell types? By cell wall angle? By length? I would like to see the authors use a more quantitative approach, here. These data are particularly important as they draw the connection between a cellular process (membrane targeting), and plant development (auxin transport/levels within the cell).

These differences were quantified taking the morphological characteristics of the caulonemata cells (more elongated cells, less chloroplast, and a diagonal cell division) compared to chloronemata cells (shorter cells, more chloroplast and perpendicular cell division). We believe that this approach is correct as we used the different morphological characteristics of the two tissues to derive the caulonemata/chloronemata ratio, as it has been reported previously (Reski R. 1998. Development, genetics and molecular biology of mosses. Botanica Acta 111: 1-15).

4) I am concerned by the fact that PINA-EGFP does not mislocalize in the cnih2 background, but rather disappears. In Supplemental Figure S5A, the authors show an additional cnih2 line with what looks to be rather normal, albeit faint, PINA- EGFP localization. If the model that the authors present is correct, then one would expect PINA-EGFP to accumulate in the ER in the cnih2 mutant (similar to what is seen in Figure 7A). Why does this not occur in the cnih2/PINA-EGFP line?

This is an observation we also made, and as the Reviewer mentions, we were expecting a signal from the ER in the cnih2 mutant. It is possible that under this condition PINA is degraded as indicated by the absence of any fluorescence. The fluorescence observed at the apex in Fig. S5 for the cnih2- 5 line is clearly lower than that observed for the wt and the cnih1-B5, as well as the fluorescence at the cell plate, which indicate that the trafficking of PINA in the cnih2-5 mutant is also affected, although not as severely as that shown in Fig. 3. The results in Fig. 7 indicate that PINA is not degraded, which could be explained by the presence of the truncated versions of CNIH2 which by interacting with the auxin transporter (See Fig. 6) seems to prevent its degradation.

5) Authors should confirm with Development team regarding policies of active/passive voice, and gene nomenclature (cornichon homologue vs CORNICHON-HOMOLOGUE).

This point has been addressed.

Reviewer 2 Comments for the Author:
The following suggestions are being made to improve the manuscript (notably to improve the reader's understanding of the rationale behind the study, and to improve the quality of the figures shown):
Title – as it stands the title is really underselling this paper. Why not be bolder – this could simply be rephrased as 'Cornichon proteins are required for the polar localization of the PINA auxin transporter in Physcomitrium patens'.

Following the Reviewer's suggestion, we have modified the title of the article.

Abstract – too much use of the word moss on line 35

The sentence has been corrected.

Introduction – generally quite comprehensive but needs to be a little more accesible

This point has been addressed.

- Line 42 – correct eucaryotic to eukaryotic

© 2023. Published by The Company of Biologists under the terms of the Creative Commons Attribution License (https://creativecommons.org/licenses/by/4.0/).

This mistake has been corrected.

- Line 52 – correct ‘has’ to ‘have’

This mistake has been corrected.
- Line 80 – ‘with no obvious phenotype’ – presumably the authors are describing the respective mutants, but this is currently unclear.

This point has been addressed.

- Line 84 – ‘pairs of AtCNIHs’ – context? Elaborate on what you mean by ‘pairs’

This point has been addressed.

- Line 99 – correct ‘cycle’ to ‘cycled’

This mistake has been corrected.

- Line 104 – vias?

This mistake has been corrected.

- Line 107 – please expand on your description of the retromer complex – what is it?

This point has been addressed.

- Line 111-113 – more explicitly needs to be explained

This point has been addressed.

- Line 123 – the authors refer to PIN gene families, but this needs to be referenced – perhaps from Viaene et al., 2014 and/or Bennett et al., 2014?

This point has been addressed.

- Line 132 (and consistently repeated thereafter) – CNIH’s – remove apostrophe please

This point has been addressed.

Results

- Lines 149 and 174 (and all other instances) – please do not refer to bryophytes as early diverging land plants – see commentary by McDaniel.

This point has been addressed.

- Line 175 – please amend hepatic to liverwort; correct ‘Sellaginella’ to ‘Selaginella’

This point has been addressed. This section has been removed as the Reviewer indicated that the data for percent identity analysis was not necessary.

- Lines 181-185 are written in an unclear manner – please rephrase

This point has been addressed.

- Line 189 – this is the first time that the authors refer to the (delta)cnih2 mutant – please can you check/confirm the conventions for the use of this terminology? I know that it is the convention for a full gene deletion – is this also the case for a partial deletion?

© 2023. Published by The Company of Biologists under the terms of the Creative Commons Attribution License (https://creativecommons.org/licenses/by/4.0/).

We have addressed this comment by modifying the corresponding text, describing the truncated CNIH1 mutant as *cnih1*, while the knockout CNIH2 mutant as *Δcnih2*.

- Line 200 – 'mutants exhibited abnormal branching' – this really isn't clear based on the figure shown. Is this the case, or do the authors indeed have better quality representative images?

We have changed the figures for the mutants to show the presence of the anormal branching, moreover, the quantification we have made (fig. 2b-c-d) help to demonstrated that these changes are significative.

- Figure S4 is completely superfluous

We would like to keep figure s4 as it shows the phenotype of the two other mutant lines that we obtained for both cnih1 and cnih2, to demonstrate that the same phenotype was observed with two additional transformation events.

-The double mutant has a 'corrected' phenotype, which is never really discussed throughout the manuscript –are CNIH1 and CNIH2 operating antagonistically? Is the cnih1 mutation dominant?

We have addressed this point in the text and agree with the reviewer, it is possible that cnih1 and cnih2 act antagonistically with cnih1 being dominat.

- MAJOR - Lines 224-226 – the authors observe that the (delta)cnih2 mutant phenotype resembles that of the pinb mutants described elsewhere. Would it therefore not have made sense to perform these studies using the PINB reporter rather than the PINA reporter? Please can the authors comment on the phenotype of the pina mutants (if known)? Some more justification is required. Also, to add to this work, could PINB not have been included in some of the protein interaction studies described later in the manuscript? It seems that PINB is entirely neglected, and it is unclear why. Perhaps the reporter line isn't very good? Please do comment.

Initially, we wanted to work with both PINA and PINB, however, after many attempts, we were unsuccessful in cloning PINB, which may be explained by the higher expression of PINA and the high similitude of the two corresponding genes (84%), which prevented us from carrying any work with this gen. Until now, a clear phenotype for the pina or pinb single mutants at any stage of the moss life cycle has not been shown, however, with the double mutant an earlier development of the caulonema was observed, which led us to carry on with our studies with the auxin transporters, although as described above, restricted to PINA as we could not cloned PINB. Because of this, we have eliminated the paragraph referring to PINB in this section. According to Viaene, T. et al 2014, both PINA and PINB reporter lines are localize at the apical and basal sides of the plasma membrane in protonemal cells but only figures from PINA reporter line was shown.

- Lines 239-242 – the ER localisation is assumed and not demonstrated – does the localisation match that of an ER marker line at the very least? I know that these are available.

We have modified the figures for the interaction between CNIH1 and CNIH2 with PINA by the BiFC to clearly show that YFP reconstitution occurred at the ER.

- Line 246 – the authors flip flop between PIN transporters and auxin transporters – please be consistent.

This point has been addressed.

- Line 248 – 'served as a negative control' – is this not simply a negative result?

This point is addressed below.

.- Supplementary Figure 5 is largely superfluous.

© 2023. Published by The Company of Biologists under the terms of the Creative Commons Attribution License (https://creativecommons.org/licenses/by/4.0/).

As it is required in the field, we generated two different mutant lines to corroborate the results which corresponds to what it is shown in Fig. S5A and prefer to keep these results as they help to support our work.

- Supplementary Figure 8 – these protoplasts do not look healthy at all, and thus nothing meaningful can be extracted from these images. Often punctate structures are formed in protoplasts – usually when they are about to explode… A nice spherical cell is required…

We repeated the transformations and obtained new images for CNIH2 wt, the CNIH2-141 and CNIH1-137 mutants showing spherical protoplasts which are now included in Fig. S11.

- Line 349 and Figure 7 – all unclear and needs an explanation

We think that our description for Fig. 7 is clear, and we have addressed the concern from the reviewer below when answering the comments to Figure 7.

- Lines 363 and 385 and Figure 7C – these images are not striking and do not really highlight the phenotypes that the authors describe as highly prevalent

We have modified Fig. 7 to highlight the morphological changes in the truncated CNIH2 mutants, where we indicate the straight growth of the protonemata in the wt, while observing undulating filaments for the two mutants. These changes are quite evident which we have observed in two transformation events for each line, that is why we are reporting them.

Methods

- Line 485 – amend ‘proportionated’ to ‘provided’

This point has been addressed.

- Line 486 – is Dra. correct?

This point has been addressed.

- Line 608 – amend ‘by’ to ‘for’

This point has been addressed.

Figures (some addressed above)
- Figure 1: Please include the protein alignment in Supplementary data. This will enable the authors to enlarge the currently very unreadable phylogeny, which can only be read on maximum zoom on screen, and even then, the support values cannot be read. Do not refer to ‘higher plants’ – it is anyway unclear whether the authors are grouping all land plants as ‘higher plants’ relative to algae. The concerns with the claims made about this tree are that the support values are too low – either include more species to resolve the tree better (and thus increase the support values) or make claims less bold.

We have included the protein alignment in Supplementary data, now denominated as Figure S1. We have changed the phylogeny to be more readable and describe only three groups. As the reviewer suggested, we included more species (around 196 species) to better resolve the tree, however, the support values were even lower than in the phylogeny of Figure 1. We think that is due to the low identity percentage between plant cornichon proteins.

- Figure 2: The graphs, in their current form, are quite unreadable and look scruffy.

This point has been addressed.

- Figure 3: The BiFC figures are barely visible. Furthermore, I am unconvinced that the authors have used the correct controls – have they considered some empty vector controls?

© 2023. Published by The Company of Biologists under the terms of the Creative Commons Attribution License (https://creativecommons.org/licenses/by/4.0/).

We do not understand what the Reviewer means with "barely visible". Previously it has been mentioned that the ER is not clearly seen, however, and to help the viewer to identify this structure, we have modified the figures to show more clearly the interaction between PpCNIH1 and PpCNIH2 with PpPINA at the ER. As for the controls, the well-known oligomerization of the aquaporins was and has been used as a positive control to demonstrate the protein-protein interaction by BiFC, that with the localization of this interaction restricted to the plasma membrane, confirms the usefulness of this control. The negative results observed with the coexpression of PpPINA with AtPIP2 indicates that although both proteins are plasma membrane located indicates that not all coexpressed proteins reconstitute YFP fluorescence. As for the use of an empty vector, we do not think this is a good control, as the expression of only half of YFP would not lead to the reconstitution of the FP, as it has been indicated (Kudla, J. & Bock, R. Lighting the Way to Protein-Protein Interactions: Recommendations on Best Practices for Bimolecular Fluorescence Complementation Analyses. The Plant Cell, 28:1002-1008, 2016).

- Figure 4: Again, the figures are unclear. Can the authors use black and white for (A) and then use different colours for (B) to make the distinction between the two lines? In the figure legend, please correct 'rigth' to 'right'.

We have made the changes suggested by the Reviewer. In the figure legend this mistake has been corrected.

- Figure 5: Unless you squint, nothing is really visible in this figure. Please also recolour to green and magenta so that the merge is more meaningful. Please check scale bar in zoomed in version – surely the scale bar needs to be altered (or removed).

In the original submission we included the original images where chloroplasts autofluorescence was subtracted to improve the signal from the fluorescent proteins as their intensity was low due to their expression been under the control of their own promoters. To help the viewer, we have increased the brightness and contrast in all figures with the same level of modification and now we think it is easy to see all the images. In the Fig. S10 we show the original images. Quantification of the co-localization corroborates that there are signals from the two fluorescent proteins.

- Figure 7 – In (A), middle image doesn't appear diffuse at all, but right-hand image does. Can the authors colour GFP and autofluorescence differently? The graph in (B) doesn't match the representative images shown in (A). I have already highlighted issues with Figure 7C above.
If we compare the ROI's from the left and central images from Fig 7A, it can be observed that the fluorescence signal from the left image is more restricted to the apex of the cell, which is associated to a higher number of pixels with low pixel intensity values (close to zero, Fig. 7B). In contrast, in the central panel the fluorescence is more diffused, and this is shown by the shift of the number of pixels towards higher pixel intensity values (closer to 255, Fig. 7B).

- Figure S1 – Serine, Threonine, Tyrosine and Threshold are not aligned with their respective coloured lines –looks odd. Figure S1c is superfluous.

This has been corrected. This figure is now Fig. S2.

- Figure S3 – exon of CNIH2 WT gene needs to be brought forward.

This point has been addressed. This figure is now Fig. S4.

- Figure S4 – earlier described as superfluous, especially if WT images aren't included alongside. I think it's OK to remove this entirely.

We have addressed the concern from the reviewer, above, when answering the comments to Figure S4. This figure is now Fig. S5.

- Figure S5 – try to improve visibility of images; in (B) please either show or describe the location of the primers used. Does this not simply show that both WT and cnih2-3 contain the endogenous

© 2023. Published by The Company of Biologists under the terms of the Creative Commons Attribution License (https://creativecommons.org/licenses/by/4.0/).

PINA gene? This (currently) does not demonstrate that the construct is contained within. Can PINA-EGFP be described as WT? I suspect that auxin transport may be perturbed in some way in this line.

Can the authors comment on this? I do think there is still merit in the results shown – clearly there is a difference between WT and the mutant lines.

We have improved the visibility of images in Fig. S5A, now Fig. S7. We show and describe in the figure legend to (B) the location of the primers used. The primers used for Fig. S5-B (Fig. S7B) were employed to corroborate that the absence of fluorescence in the cnih2 mutant was not due to an alteration in the PINA-GFP expression, therefore, the absence of fluorescence in the cnih2 mutant is mainly due to a defect in the trafficking of the PINA protein. Yes, the results in Fig. S5B do confirm that both lines, wt and Δcnih2-3 mutant in the PINA-GFP reporter line contain the endogenous PINA gene.
Confirmation that the construct is contained in the PINA-GFP line was shown in the original article (Viaene, T. et al.2014. Directional auxin transport mechanisms in early diverging land plants. Current Biology 24, 2786-2791). We agree, we have changed the WT label to PINA-GFP. According to the report where this line was characterised, the morphology of the moss was not affected. This construct is under the control of the PINA promoter.

- Figure S6 – please amend HR schematic. It is usually convention to show the construct recombining with the endogenous WT locus, and then the final recombined locus (i.e., put the construct at the top of each)

We have made the changes suggested by the Reviewer. This figure is now Fig. S8.

- Figure S8 – I have already commented on this above. The protoplasts are not of sufficient quality to make any bold claims. This looks like an artefact.

We have changed the images showing healthy spherical protoplasts. This figure is now Fig. S11.

<u>Second decision letter</u>

MS ID#: DEVELOP/2023/201635

MS TITLE: A cornichon protein controls polar localization of the PINA auxin transporter in Physcomitrium patens

AUTHORS: Carolina Yanez-Dominguez, Daniel Lagunas-Gomez, Diana Milena Torres-Cifuentes, Magdalena Bezanilla, and Omar Pantoja
ARTICLE TYPE: Research Article

Dear Dr. Pantoja

I am happy to tell you that your manuscript has been accepted for publication in Development, pending our standard ethics checks. As you will see, the reviewer points out that your results may indicate that the truncation allele may be acting as a dominant negative--I think this is possible and recommend adding a line in the discussionto this point

<u>Reviewer 1</u>

Advance summary and potential significance to field

Aside from occasional spelling and grammar issues, the authors have satisfied the issues I raised in my initial review.

© 2023. Published by The Company of Biologists under the terms of the Creative Commons Attribution License (https://creativecommons.org/licenses/by/4.0/).

Comments for the author

I appreciate the authors' timely response to my recommendations. My main criticism is with regards to the differences between PINA-GFP localization in the mutant background versus the truncated complementation experiment. Looking back through, it occurs to me that the truncated protein may be having a dominant negative effect. The authors may want to consider that, and include such language in their discussion if they so chose.

© 2023. Published by The Company of Biologists under the terms of the Creative Commons Attribution License (https://creativecommons.org/licenses/by/4.0/).
